# Climate Warming and Seasonal Precipitation Change Interact to Limit Species Distribution Shifts across Western North America

**DOI:** 10.1371/journal.pone.0159184

**Published:** 2016-07-22

**Authors:** Melanie A. Harsch, Janneke HilleRisLambers

**Affiliations:** Department of Biology, University of Washington, Seattle, WA, United States of America; Ecole Pratique des Hautes Etudes, FRANCE

## Abstract

Using an extensive network of occurrence records for 293 plant species collected over the past 40 years across a climatically diverse geographic section of western North America, we find that plant species distributions were just as likely to shift upwards (i.e., towards higher elevations) as downward (i.e., towards lower elevations)–despite consistent warming across the study area. Although there was no clear directional response to climate warming across the entire study area, there was significant region- to region- variation in responses (i.e. from as many as 73% to as few as 32% of species shifting upward). To understand the factors that might be controlling region-specific distributional shifts of plant species, we explored the relationship between the direction of change in distribution limits and the nature of recent climate change. We found that the direction that distribution limits shifted was explained by an interaction between the rate of change in local summer temperatures and seasonal precipitation. Specifically, species were more likely to shift upward at their upper elevational limit when minimum temperatures increased *and* snowfall was unchanging or declined at slower rates (<0.5 mm/year). This suggests that both low temperature and water availability limit upward shifts at upper elevation limits. By contrast, species were more likely to shift upwards at their lower elevation limit when maximum temperatures increased, but also shifted upwards under conditions of cooling temperatures when precipitation decreased. This suggests increased water stress may drive upward shifts at lower elevation limits. Our results suggest that species’ elevational distribution shifts are not predictable by climate warming alone but depend on the interaction between seasonal temperature and precipitation change.

## Introduction

Many studies document distribution shifts of plants, suggesting that distribution limits are determined by climatic factors [1] and that species are already responding to changes in these factors [2, 3]. In general, documented and projected distribution shifts are towards the poles or higher elevations, with high species-to-species variability in responses [3–6]. Efforts to model the effect of recent and projected climate change on species persistence have focused on species’ ability to track shifting climatic regimes at the upper elevational or latitudinal distribution limit [7–9]. However, focusing solely on upward shifts (toward poles and higher elevations) at the distribution limit misses the fact that many species distributions are either not shifting [6] or are shifting downward [2, 3, 10, 11].

The effect of climate change on future species’ distributions is likely to be complex, given the potentially differing climatic controls over upper and lower distribution limits. There is strong evidence that minimum temperatures consistently determine upper elevational and latitudinal distribution limits [12], but lower distribution limits are thought to be determined by an interplay of temperature and water availability [10, 13–15]. Water limitation at lower distribution limits has been suggested by model simulations [15] and field studies [10, 14]. Climate change influences average water availability through warmer summer temperatures and changing rainfall patterns [16] as well as by affecting snowfall and snowpack dynamics, which influence water availability in summer dry systems [17, 18]. Given that regional warming is not necessarily correlated with changes in water stress, which is increasing in some cases and decreasing in others [19], distribution shifts in response to recent climate change could therefore occur in either direction (upward or downward).

**Fig 1:**
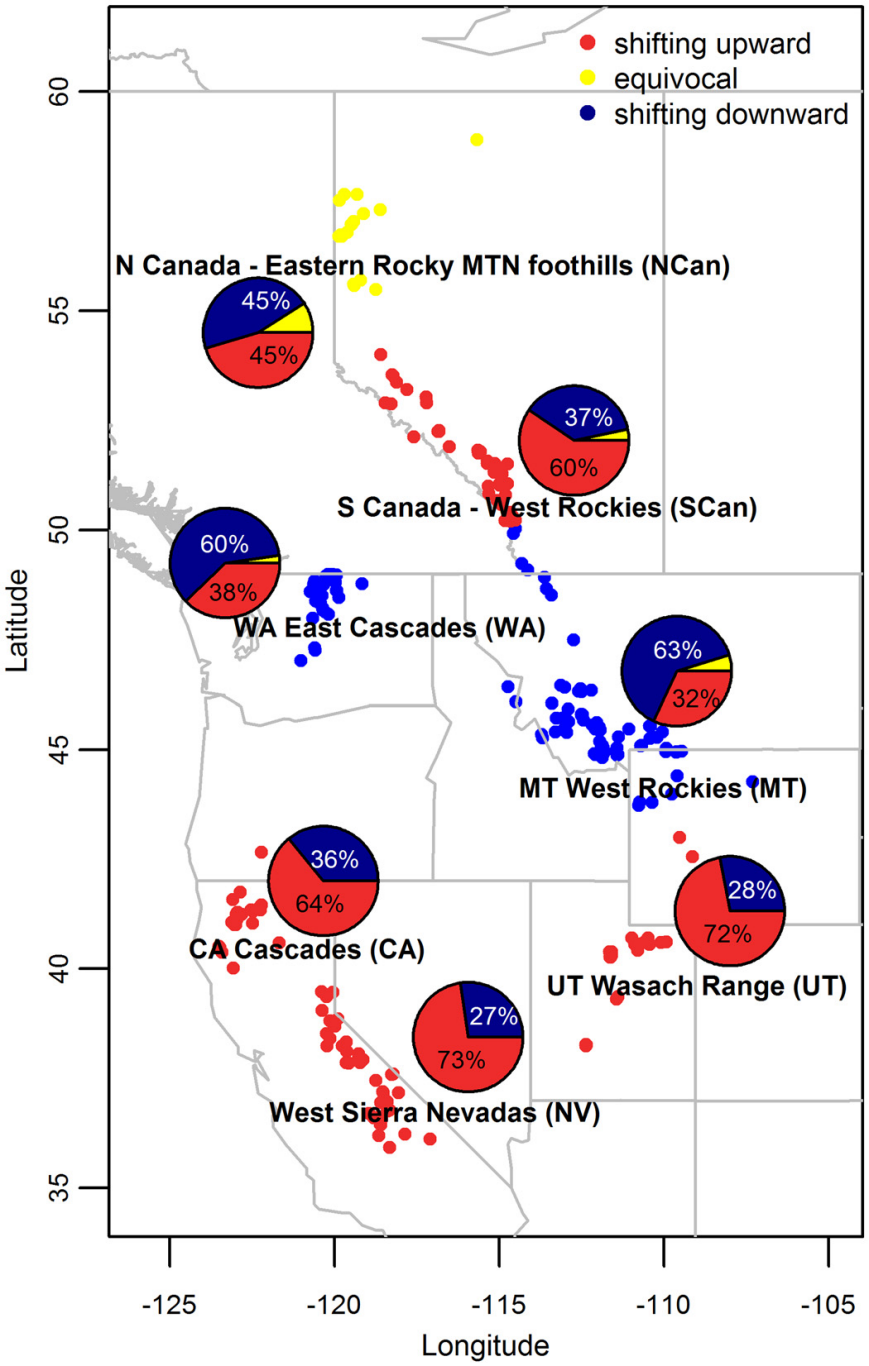
Species distribution shifts were calculated within each of the seven topographically distinct regions within western North America. Each circle represents the geographic coordinate of the highest elevation of occurrence for each species within a region. All circles within regions are colored according to whether most species (Binomial test, p<0.05, see Table 1) in that region shifted upward (red), downward (blue), or if the direction of change was equivocal (Binomial test, p > 0.05) (yellow). Pie charts indicate the percentage of species shifting in each direction within each region (see Table 1).

We use species occurrence records of 293 plant species within seven regions (444 species-region combinations) across western North America (Fig 1) to evaluate 1) whether plant species have consistently responded in the expected direction (an upward shift at the distribution mean) to 40 years of climate change across the entire study area; 2) how region and lifeform influence the direction of species distribution shifts and proportion of species responding; and 3) whether the critical climatic factors correlated with species distributions shift differ between upper and lower distribution limits. Addressing these objectives allows us to speculate whether or not plant species are likely to respond in a consistent manner (upward or downward distribution shifts) to further climatic changes over the next 40 years across this broad geographic area.

## Materials and Methods

### Data sources

Vegetation occurrence records (presence-only) were downloaded from five databases, USDA Forest Inventory and Analysis (http://apps.fs.fed.us/fiadb-downloads/datamart.html), VegBank (http://vegbank.org), the Government of Canada Ecological Site Information database (http://srd.alberta.ca/MapsPhotosPublications/Maps/ResourceDataProductCatalogue/Biophysical.aspx), CalFlora (www.calflora.org), and the Global Biodiversity Information Facility (www.gbif.org) between January and February 2014. Further details on the individual databases are provided in S1 Appendix. Records were available for 293 species: 41 tree species (adult and seedling occurrences), 51 shrub species, 173 herbaceous plant species, and 29 grass and sedge species. Databases were comprised of observation data collected using field-based methods such as species presence within plots or transects (3 of 5 databases) or a mixture of historical records and observational data (2 databases; CalFlora, Global Biodiversity Information Facility). In all cases, field-based records were described as being collected “systematically” and “repeatedly”. Further references to the databases will be in regards whether the data are based solely on field-based records (field-based) or a mixture (historical).

Publically-available data sources, although an incredibly rich resource, have drawbacks. Temporal and spatial biases are expected in museum collections but are also present in field-based studies (S1 Fig). Identifying collection biases in historical records and observational data is a challenge. Documentation can indicate certainty in occurrence records, but only if reported and in a meaningful way. Methods have been developed to correct for potential collection biases [2, 20, 21], including null model correction, the approach taken in this paper (methods detailed below and in S1 Appendix, results shown in S2 Appendix).

An additional question is whether it is appropriate to merge such disparate data sources. We argue that it is for this analysis, as long as data are simplified to the lowest common denominator across databases: occurrence records. Valuable data present in some databases but not others (i.e. absences and density) is lost, but at the benefit of greater temporal depth. Even after merging, the question remains as to whether some data sources should be given greater weight for inference than others [21]. As discussed in S2 Appendix, these weighting approaches should be viewed with caution. In this study, the aggregated dataset show less temporal fluctuation in elevational sampling effort than the individual pieces (S2 Fig). In summary, we chose to use unweighted data because weighting data sources is unlikely to be a simple, straight-forward process and would require considerable thought and methodological development beyond the scope of this study.

The bounty of data becoming publically available represents a valuable resource that will provide much greater temporal and spatial scope than traditional data sources. Developing standard protocols and clear methodologies is a critical step for utilizing this valuable resource. Because we felt we did not have the information to appropriately weight data sources, we instead quantify and attempt to remove collection bias. First, we carefully consider the datasets comprising each database and remove as much uncertainty from each as possible. For instance, some databases provide information on whether individual datasets are validated or reliable. Individual datasets within databases with low certainty were removed. An additional uncertainty, sampling bias, requires additional evaluation. Visualizing the data show that sampling biases are present in all datasets (S2 Fig and Fig B in S2 Appendix) but that incorporating data from multiple databases can reduce sampling bias (S2 Fig and Fig B in S2 Appendix). Even after merging multiple data sources, sampling biases may still be present. To account for this bias, we use a null model approach. Details on this approach are provided below in the *Assessing directional changes in species distributions* section and in S2 Appendix.

### Data processing

Occurrence records were excluded if species name, year, or geographic collection-coordinate (latitude, longitude) were missing or uncertain, based on notes within the database, or if geographic collection coordinate precision was less than 50 m. Coordinate position for occurrences in all databases (but not necessarily all datasets within a database) are intentionally fuzzied and the degree of fuzzying is not reported. Uncertainty in plot locations is thus inevitable for such datasets. However, as the direction and distance of such prescribed measurement error is random, it should not influence the direction of shift quantified across all species. If fuzzying has any effect on our results, it more likely decreases our ability to detect a shift for any one species than driving the direction (or magnitude) of average shifts across all species or groups of species within a region. We evaluate whether coordinate error along with temporal and spatial sampling bias affected distribution shift estimates in S2 Appendix.

Duplicate records across all databases, based on species name, collection coordinate, and year, were removed. To fill in missing elevation records and correct elevation records reported in feet, rather than meters, above sea level, we estimated altitude using satellite data for all occurrences using the GNsrtm3 function within the geonames package [22] implemented in R v3.1 [23]. The accuracy of estimating elevations was evaluated by regressing reported elevations (missing values removed but not values reported in feet above sea level) against estimated elevation using the GNsrtm3 function, and was found to be very high (linear regression, intercept = 166.2, slope = 0.9189, t = 811.56, R^2^ = 0.87, p < 0.05).

Misspelling of species’ names was manually checked for and corrected. We followed specifications within each database for confidence in taxonomic names and how to correct for changes in taxonomic usage between years. Another potential source of error is taxonomic nomenclature. Databases follow a standard protocol for nomenclature (USDA Forest Inventory and Analysis and the Government of Canada Ecological Site Information database) or employ a standard procedure to ensure consistency in nomenclature within their database (VegBank). In all cases, the databases aimed to map historical names to current names either through algorithms employed within the database or during data processing. To further ensure consistency between databases, taxonomic standardization was validated using the Taxonomic Name Resolution Service v3.2 [24]. One species in our database was flagged and resolved.

### Regions

To take into account climatic and topographic variability, the study area was divided into seven regions based on ecoregions as defined within Omernik (2004). These ecoregions are differentiated based on slope (east or west), soils, hydrology, vegetation, and climate (temperature and precipitation) [25]. We do not use ecoregions to make inferences about how species within ecoregions will respond to climate change, but, rather, to objectively group occurrence records around mountainous areas. Subsequent data processing and analyses are for species within regions. We restricted our analysis to species within regions meeting the following criteria: at least 50 occurrence records per species within a region (median = 122, mean = 189, max = 816), minimum observation year 1979 or earlier, maximum observation year 1995 or later, and at least 10 occurrence records for each decade of analysis.

### Assessing directional changes in species distributions

We examine the direction of elevational shifts at species distributional means and distribution limits. We first estimate the raw annual shift rate at the distribution mean for each species within each region as the slope of the least squares regression line for the relationship between year and the recorded occurrence elevation. Raw shifts at the distribution limits were calculated using quantile regression, specifying the 5th and 95th quantiles for the lower and upper elevational limits, respectively (code provided in S3 Appendix). As 108 of the 293 species occurred in multiple regions, we were able to estimate shifts at the distribution mean, and the upper and lower distribution limit for 444 species-region combinations. Although individual plants may have been observed multiple times, we cannot tell, from the data provided, which records come from the same plant. Such observations (as well as observations made in the same location multiple times) could introduce temporal autocorrelation into our results. To address this issue, we test for autocorrelation in our dataset using the Dublin Watson test. Of the 444 species-region combinations, 15 showed signs of autocorrelation (p < 0.05), and half of these species (7) had autocorrelation values less than 0.5.

Temporal biases in elevation sampling effort may be present in datasets that utilize online occurrence records. We use a null model approach to assess whether this bias is present and account for it (code provided in S3 Appendix), by calculating a null shift rate for each species within each region at the distribution mean [20]. The null shift rate analysis was conducted using the unprocessed dataset (species with insufficient number of observations included). Specifically, we took the following steps for each species within our post-processed data: 1) subset the unprocessed dataset to all records (all species) within the region that the focal species occurred, 2) further subset to include only records within the minimum and maximum observation of occurrence for the focal species (across all time periods), 3) randomly select occurrence records (based on the number of occurrence records for the focal species), and 4) obtain the slope for the relationship between year and the recorded occurrence elevation. This approach allows us to assess, for each focal species in the region of interest, whether sampling efforts across elevation have changed over time. We repeated these steps 500 times for each species-region combination, and obtained the null shift rate from the mean of the 500 simulations. This procedure therefore allowed us to calculate null shift rates for each species-region combination within the dataset. We can then account for temporal sampling bias by subtracting the null shift rate from the raw shift rate. This is the corrected shift rate. For shift rates at the upper and lower limit, we subtract the null shift rate from the raw shift rate calculated using quantile regression. Throughout the manuscript, reported shift rates are these corrected shift rates—i.e. raw shift rates with null shift rates subtracted.

### Assessing the proportion of upward distribution shifts within regions

We assess whether the proportion of species shifting upward at the distribution mean significantly differs from the proportion of species shifting downward using a binomial test across the entire study area as well as within regions. We class species as having shifted in distribution (upwards or downwards) not based on significance of the linear model to obtain raw shift rates but based on whether corrected shift rates would be readily detected within the timeframe of this study (≥0.25 m/yr or <10 m in elevation over 40 years). We do this for two reasons: 1) because we are using corrected shift rates and 2) because no response (not shifting) and slow shift rates are as informative as statistically significant shift rates. Species with shift rates less than 0.25 m/yr were classed as not shifting and are reported in Fig 1 and Table 1 but not included in analyses of binary directional shifts (upward or not). Proportional changes in the direction that species distributions shifted were calculated at the distribution mean by classing estimated shift rates as being upward (positive slopes) or downward (negative slopes). We also assessed whether certain lifeforms were more likely to shift upward at the distribution mean using ANOVA.

**Table 1:** Percent of species within regions exhibiting upward shifts at the distribution mean, lower distribution limit, and upper distribution limit.

	NCan	SCan	WA	MT	CA	NV	UT
**Proportion shifting upward at distribution mean, upper and lower limits**							
Mean	45.5	59.6	37.8	32.0	64.1	72.7	72.0
Upper limit	50.9	26.9	60.0	25.6	59.0	74.2	64.0
Lower limit	3.6	93.3	37.8	56.0	66.7	81.8	56.0
**Net change across a species’ distribution (upper + lower distribution limits)**							
upward	1.8	25.8	20.0	17.6	38.5	57.6	32.0
expansion	56.4	1.1	40.0	10.4	20.5	16.7	32.0
downward	40.0	2.2	22.2	32.0	12.8	1.5	12.0
contraction	1.8	70.8	17.8	40.0	28.2	24.2	24.0
**No. of species**	**55**	**89**	**45**	**125**	**39**	**66**	**25**

Result for distribution limits are based on quantile regression for species within regions with at least 50 observations. The lower distribution limit reflects the 5^th^ percentile and the upper distribution limit the 95^th^ percentile. We also show the net change across species distributions as the percent of species within a region exhibiting upward shifts (lower and upper distribution limits shift upward), expansion (lower limit shifts down and upper limit shifts upward), downward (lower and upper limits shift downward), and contraction (lower limit shifts upward and upper limit shifts downward). Abbreviations for the regions are NCan: Northern Canada Rockies, SCan: Southern Canada Rockies, WA: Washington Cascade region, MT: western Rockies around Montana, CA: Cascade Range within California, NV: Sierra Nevadas, UT: Wasach Range in Utah.

We checked for possible errors in our estimated shift rates due to land-use and urbanization and data processing choices. Apart from the Utah Rockies region, urban centers with populations greater than 100,000 were not near any of the species occurrences, regardless of decade. In addition, 99.6% (117,037/117,560) of all raw occurrence records were above the mean elevation for urban centers included in the study (457 masl). No effects due to data processing choices were evident (see S1 Appendix).

### Determining local climatic change

Our analysis of the effects of climate change on distribution shifts focuses on climatic conditions at the distribution limits rather than the distribution mean because 1) species’ sensitivity to climatic conditions should be greater at distribution limits than at distribution means [26] and 2) upper and lower distribution limits are expected to respond to different climatic drivers. Rather than using climate data from a single point of occurrence (highest or lowest elevation of occurrence), we used the mean climate data across occurrence locations at the upper and lower distribution limits, respectively.

Climate data was obtained from the ClimateWNA database [27] for the period 1960–2009 for each species-region combination. Climate data within the ClimateWNA database (originally derived from a gridded product—PRISM [28]) are downscaled to a point location using elevation from a DEM model for the reference period (1961–1990). Climate data outside the reference period (1991–2009) are generated by integrating with downscaled data from the reference period [29, 30]. Although we would ideally relate species-specific distributions to local climate data, these data are not available for the majority of our species-region combinations, and thus, scale-free climate data is the best available data for local climate analyses.

For each-species region combination, we downloaded climatic conditions for the highest and lowest 10% of occurrence records (in masl), respectively. Using this data, we calculate the mean climate data at upper and lower distribution limits across the study period (1960–2009) and the rate of change in climate variables for each species-region combination at the lower and upper distribution limits. We focus on climate variables that affect growing season length (minimum summer and winter temperature, precipitation falling as snow—snowfall) and water-stress (potential evapotranspiration, rainfall, and maximum summer temperature). Of course, factors like soil quality can modify impacts of climate factors on soil moisture availability; these data are not widely available and incorporating them into analyses is nontrivial.

We determined rates of local climatic change in climate variables by calculating the mean annual rate of change in each climate variable over the period 1960–2009, the slope of the least squares regression line for the relationship between the seasonal climate variable and year. This was the same method used to calculate distribution shift rates. For minimum and maximum temperatures, we used the seasonal minimum and maximum temperatures, respectively (S3 and S4 Figs). We included climate data for the ten years prior to the earliest occurrence record to take into account any potential lags in species’ response to climatic changes. Thus, these models fit the overall trend that climatic variables are changing but do not capture inter-annual variability (S5 Fig), as precision in occurrence records is insufficient for analyzing response to inter-annual climate variability.

### Assessing climatic effects on directional shifts

To assess how strongly local climatic change influences shift directions at upper and lower distribution limits, we evaluated the relationship between the rate of change in climatic variables and distribution shift direction (S1 Table). We did so in the following steps. First, we classed direction of change as a binary variable (with species shifting either upward or not). We consider shift rates that would not be readily detected within the timespan of this study (<0.25; <10 masl/40 years) to be equivocal (not moving upward or downward). We therefore class directional response as upward (shift rate > 0.25) or not (shift rate < 0.25). Second, we evaluated the influence of each explanatory variable (rate of change for each climatic variable) alone on distribution shift direction in univariate models. Finally, we developed a multivariate generalized linear model (using a binomial distribution) that only included the seven explanatory variables that were significant in these univariate models (p < 0.05). As no response (not shifting) and slow shift rates are as informative as statistically significant shifts in distribution, we included all species in analyses, regardless of the significance of the estimated shift rates. Several species occurred in multiple regions, which might result in correlated responses, but we did not include a species-level random effect in the model because most species (175 of 293) occurred in only one region.

Prior to model formulation, all seven explanatory variables (the rate of change in average winter and summer minimum and maximum temperature—four potential explanatory variables, total summer rainfall or winter precipitation falling as snow—two potential explanatory variables, and climate moisture deficit—one potential explanatory variable) were evaluated for independence. If variables were correlated (r > 0.6, S1 Table), we selected the group of explanatory variables that were most highly correlated with response variables (S2 Table). Explanatory variables were then standardized by subtracting the mean and dividing by the standard deviation [31] to put all variables on the same scale. The best multivariate models (1 each, high and low elevations), developed for the entire study area and including region as a random effect, were selected according to change in AIC values (S3 Table). Interactions were considered between the rate of change in seasonal precipitation variables (rain or snow) and summer temperature extremes and were including if interactions improved the model (determined by a > 2 reduction in AIC value) over the best model without interactions. Code for climate analysis is provided in S3 Appendix.

We next ran a similar analysis for continuous shift rates, where we address the question of whether the shift rate at the distribution limits is related to climatic change, and if so, which climatic variables. In this analysis, we assumed a Gaussian error distribution. As results were qualitatively similar between the direction of change and shift rate analysis (S4 Table), we present results for the shift direction at the distribution limits. All analyses were conducted within R 3.1 [23].

## Results

**Fig 2:**
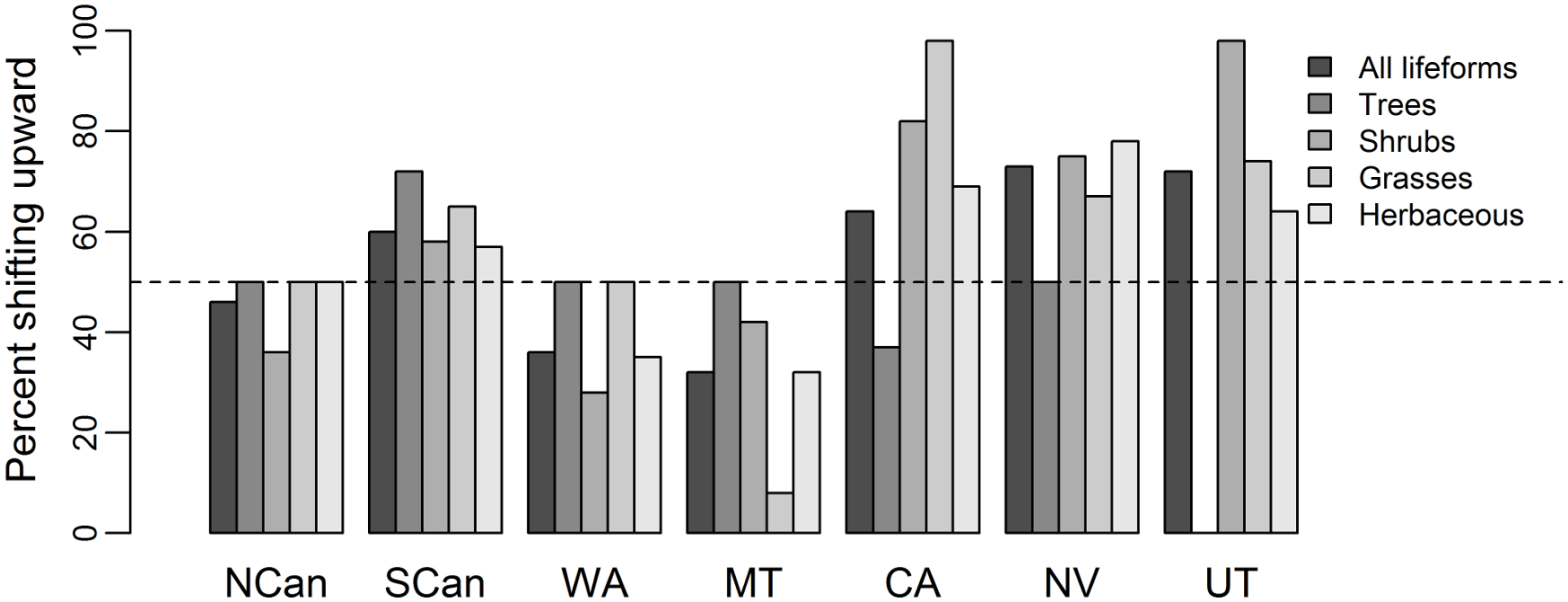
Percentage of species exhibiting upward elevational shifts at the distribution mean over the time period 1970–2009 across western North America across four lifeforms, and for each lifeform: trees, shrubs, grasses and sedges, herbaceous plants. Abbreviations for the regions are NCan: Northern Canada Rockies, SCan: Southern Canada Rockies, WA: Washington Cascade region, MT: western Rockies around Montana, CA: Cascade Range within California, NV: Sierra Nevadas, UT: Wasach Range in Utah.

Across western North America, species within regions were just as likely to exhibit upward elevational shifts at the distribution mean (50.9%, 226/444) as they were downward elevational shifts (45.3%, 201/444; Binomial test, p > 0.05) (Table 1, Figs 1 and 2). Upper distribution limits shifted in the same direction as the mean of the distribution (≥55% of species shift upward or downward in both positons along the distribution) in 5 of the 7 regions, whereas lower distribution limits shifted in the same direction as mean distributions shifts in 4 of 7 regions. In general, upward shifts were more prevalent at lower elevation limits (59%; 264/444) and distribution means (50.5%) than at upper elevation limits (45%; 198/444). Regional differences in the direction that distribution means shifted were evident. On average, upward shifts were evident within the Southern Canada Rockies, the Cascade Range within California, the Sierra Nevadas, and the Wasach Range in Utah (59–73% of species shifting upward; Binomial test p < 0.05). In contrast, most species shifted downward at the distribution mean (60%-63%; Binomial test p < 0.05) in the western Rockies around Montana and the Washington Cascades region. A consistent shift direction at the distribution mean (Binomial test, p > 0.05) was not evident for the Northern Canada Rockies (Fig 1, Table 1).

Species occurring in multiple regions tended to follow regional patterns rather than exhibiting species-specific patterns. For example, looking across regions with divergent overall directions of change, 82% (28/34) of the species occurring in at least three regions followed regional trends but only 18% (6/34) exhibited consistent species-specific responses. In addition, the direction that the distribution mean has shifted is, in general, not related to lifeform. We found no indication that certain lifeforms are more likely to shift upward at the distribution mean than other lifeforms (ANOVA, F-value = 1.009, p-value > 0.05; Fig 2). Specifically, trees, shrubs, grasses and sedges, and herbaceous plants tended to exhibit consistent patterns in the overall regional shift direction (Fig 2). There was some variation within regions for life-form specific results. For example, all lifeforms tend to shift upward in the Cascade Range within California except trees (36% of tree species shifting upward). However, there was no indication that some lifeforms were more likely to shift upwards than other lifeforms.

The direction of distribution shifts could not be explained by temperature changes alone. Winter warming was evident for all species at upper distribution limits (99% experiencing warmer temperatures; Table 2). Most species also experienced warmer summer temperatures (Table 2)–maximum summer temperatures have warmed for 71% of species at high elevation limits (306/430) and minimum summer temperatures have warmed for 77% of species at high elevation limits and 83% of species at low elevation limits (333/430; 357/430 climate data was not available for 14 species). However, despite consistent warming, only 45% (198/444) of the species exhibit upward shifts at the upper distribution limit and 59% (264/444) at the lower distribution limit. Warming maximum summer temperatures were less prevalent at lower limits (60%; 265/444) and not a significant predictor, on its own, of the direction that distributions shifted at lower limits (linear model, t = -1.004, p > 0.05).

**Table 2:** Proportion of species that experienced warming or increased precipitation during the summer and winter months between 1960 and 2009 at low and high elevation limits.

	Summer	Winter
**Low elevation limit**		
** Maximum Temperature**	**71.3**	**98.6**
** Minimum Temperature**	**77.5**	**35.2**
** Rainfall**	**73.4**	
** Snowfall**		**7.8**
**High elevation limit**		
** Maximum Temperature**	**59.0**	**93.5**
** Minimum Temperature**	**82.9**	**100**
** Rainfall**	**92.4**	
** Snowfall**		**0**

**Fig 3:**
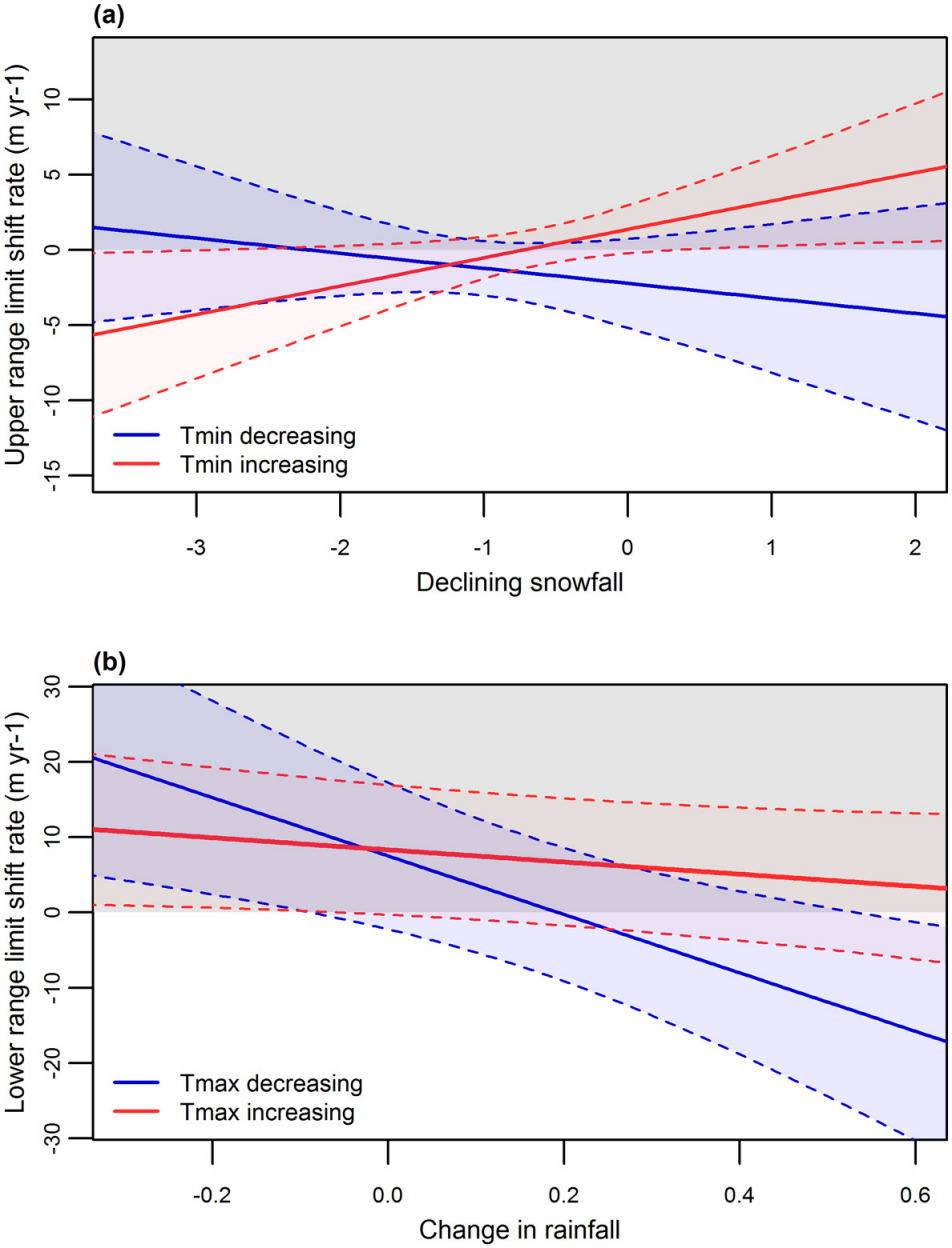
Mean (solid lines) and 95% confidence intervals (dashed lines) for the modeled relationship between shift rates at (a) high elevation distribution limits and snowfall decline (mm yr^-1^) and (b) low elevation distribution limits and change in rainfall (mm yr^-1^) when the maximum summer temperature has warmed (Tmax increasing) or cooled (Tmax decreasing). The grey shaded area represents the where distribution shift rates are positive and distribution limits are shifting upward.

We found that considering how summer temperature and seasonal precipitation changed jointly provided better insight into how distributions shifted than the rate of change in temperature alone. The effect of summer warming and seasonal precipitation change depended on the distribution limit being considered (Table 3). For example, when minimum summer temperatures are warming, upward shifts at high elevation distribution limits were more likely to occur as long as the amount of precipitation falling as snow is declining less rapidly (nearly all species experienced declining snowfall, but the magnitude of declines varied greatly) (Table 3, Fig 3). Although downward shifts were just as likely to occur when summer rainfall declined (73%) as when winter snowfall declined (60%), the effect of summer rainfall change was not significant in the multivariate model. At low elevation distribution limits, there was an interaction between warming and precipitation changes, with species distributions more likely to shift upward when maximum temperatures increased regardless of rainfall, but also shifted upward under cooling temperatures when summer rainfall decreased (Table 3, Fig 3). The rate of change in summer temperature and precipitation change (snowfall at upper elevation limits and rainfall at lower elevation limits) was not well correlated at upper (0.53) or lower (-0.04) elevation limits (S1 Table). In summary, in high elevation sites with warming minimum summer temperatures, species were more likely to shift upward when winter snowfall declined less (Fig 3a). In low elevation sites with warming maximum summer temperatures, species were more likely to shift upward when summer rainfall decreased (Fig 3b).

**Table 3:** Coefficients from best-fitting linear models testing which local climatic changes (over the past 50 years) explain the direction (upward or downward) of elevational distribution shifts at high and low elevation limits over the last 40 years.

	High elevation limit			Low elevation limit		
	Estimate	SE	z-value	Estimate	SE	z-value
Tmax				0.244	0.176	1.387
Tmin	0.539	0.228	2.366			
Rain				-1.053	0.325	-3.421
Snow	-0.438	0.190	-2.310			
Tmin*Snow	0.558	0.156	3.566			
Tmax*Rain				0.471	0.195	2.412
Water stress						

Best fitting models were selected from multiple models using AIC values (S2 Table), and included region as a random effect, the annual rates of change for summer mean maximum (Tmax) and minimum (Tmin) temperatures, mean summer precipitation (Rain) and mean winter precipitation as snow (Snow), along with an interaction between the annual rate of change in the maximum summer temperature and precipitation as snow, and mean water stress over the study period measured as Hargreaves climate moisture deficit. Winter warming was not included as all species experienced warming minimum temperatures at upper distribution limits (430/430) and nearly all species experienced warmer maximum temperatures at lower distribution limits (426/430). All climatic variables are significant to p < 0.05. Note that climate data was not available for 12 species-region combinations.

## Discussion

Across western North America, species distributions are shifting in response to climate change, but in a way that suggests factors in addition to warming are important. Given the wide prevalence of climate warming in either minimum or maximum temperatures (84% of species experienced summer warming in either minimum or maximum temperatures and all species experienced winter warming at the high elevation distribution limit), we expected that the majority of species would exhibit upward elevational shifts. Yet, only half of the species in our dataset are shifting upward. It is possible that the frequency of upward shifts did not meet expectations because we assessed shifts at upper and lower distribution limits along with the distribution mean. We expected the frequency of upward shifts to be greatest at upper elevation limits and lower at the distribution mean and lower elevation limit. This is because sensitivity to climatic conditions tends to increase from the distribution mean to the distribution limits [26]. Also, low temperatures are expected to limit upper elevation distribution limits more so than lower elevation distribution limits [26]. Contrary to this expectation, we did not find greater occurrence of upward shifts at upper elevation limits compared with lower elevation limits or the distribution mean. Upward shifts were more prevalent at lower elevation limits (59%; 264/444) and distribution means (50.5%) than at upper elevation limits (45%; 198/444).

Why are just as many species’ distributions shifting to lower elevations as to higher elevations? Neither lifeform (Fig 2) nor lack of climate warming appears to be driving this pattern. Instead, the different distributional shifts we observed are best explained by how temperature and precipitation have both changed. Downward shifts at the high elevation limit tended to occur with more rapid rates of decline in snowfall, whereas precipitation increases were associated with downward shifts at low elevation limits (Table 3, Fig 3). The effects of precipitation change thus interacted with warming rates, but in a different way at upper vs. lower distribution limits (Fig 3). For example, upward shifts were most likely at high elevations when minimum summer temperatures warmed and snowfall increased, whereas upward shifts were most likely at low elevations where maximum summer temperatures increased and rainfall decreased.

Snow and precipitation can constrain distribution limits and influence distribution shifts in many ways. For example, declining snowfall could lead to longer growing seasons, facilitating upward shifts at upper distribution limits [32], where short growing seasons constrain growth [33, 34]. On the other hand, declining snowfall could increase exposure to early season frosts or increased soil freezing depth, resulting in dieback and mortality at the upper distribution limit. Declining snowfall could also influence water availability (through snowmelt [35, 36]) at critical life-history stages (e.g. seedling germination and flowering) in spring or early summer, and may make plants less tolerant to drought and extreme temperature [37]. Because we found that species were just as likely to shift downward when summer rain declined as when winter snowfall declined (73 vs. 60%), we believe that the effects of declining snowfall on water availability played a stronger role than its effects on growing season length or frost damage [17, 18]. Thus, our results suggest that changes in winter snowfall mediate the importance of summer drought, with declining snowfall potentially exacerbating summer warming and limiting upward distribution shifts.

We also found that changes in water availability likely influence distributions shifts at lower distribution limits. Here, we found that upward shifts were most likely when rainfall was decreasing and downward shifts were most likely when rainfall was increasing (Fig 3), implying water stress. Increased water stress has been shown to increase tree mortality rates [38], potentially leading to range contraction at low elevation limits (upward distribution shifts). The idea that water stress plays an important role is also supported by our finding that species experiencing declining maximum summer temperatures are more likely to experience downward distribution shifts if also experiencing increasing rainfall. Presumably, increased precipitation along with decreased temperatures leads to decreased water stress at all elevations, allowing low elevation limits (if limited by water availability), and, consequently, the distributional mean, to shift downward [2]. Change in precipitation, although variable, are projected to intensify with climate change, with wet areas getting wetter and dry areas getting drier [39, 40]–potentially influencing future distribution shifts even more.

Determining the underlying mechanisms by which climate change is driving the distribution shifts, like the one observed in this study, is important for developing predictions of the generality and longevity of both upward and downward distributions shifts. Although this study highlights the importance of changes in both temperature and precipitation in influencing distribution shifts, it does not provide insight on whether these variables directly or indirectly drive distributions shifts. This is important, since distribution shifts driven by direct effects of climate on reproduction and survival may be expected to respond in synchrony (with potential time lag) with climatic changes. In contrast, distribution shifts driven by indirect effects of climate change (i.e., changes in competitive ability) may be more idiosyncratic and harder to predict temporally and spatially. Biotic interactions are often assumed to play a larger role at lower distribution limits, although few studies have tested this directly [41, 42] and a recent literature review found that abiotic factors more often explained lower distribution limit positions than biotic factors [43]. Although we cannot rule out the potential for biotic interactions to be driving the direction that species distributions have shifted, the consistent response across species and lifeforms to regional changes in climate suggests that distribution shifts may be driven more by direct than indirect effects of climate change. However, additional studies would be needed to fully assess these possibilities.

In summary, our results highlight the complexity of climate change-induced distribution shifts. First, distribution shifts vary by region, with trees, shrubs, grasses and sedges, and herbaceous plants within regions shifting in a consistent manner. Second, the critical climatic factors associated with upward vs. downward species distributions shifts differed. These two results suggest that local climate is driving the net direction of change in species distributions, not life-form differences across regions. Third, we find that plant distribution shifts in western North America are explained by changes in both summer and winter climatic variables, sometimes in complex interacting ways. Specifically, declining snowfall at high elevation limits appears to be mediating the distributional responses of species to climatic changes during the growing season [44, 45]. If Western North America experiences further warming and region-specific changes in snow and rainfall [46], we would expect upward distributional shifts with continued species-to-species variability driven by water availability.

## Authors Comments

This manuscript was previously published by Global Change Biology [47]. The article was retracted for the following reason: one approach used to account for sampling bias, the null model approach, was affected by a coding error. This coding error affected the presented broad-scale patterns of species distribution shifts in response to recent climate change. In the retracted article, we report that 60% of species distributions had shifted downward. In this revised manuscript with corrected null model, we report that 45% of species distributions shifted downward. All other presented results reflect quantitative, rather than qualitative, changes. The manuscript has been revised to focus on examining potential causes of the direction that distribution shifts rather than examining downward shifts.

## Supporting Information

S1 Appendix Supplementary methods. (DOCX)

S2 Appendix Additional details on bias analysis. (DOCX)

S3 Appendix R Code for data analysis. (R)

S1 Data Occurrence records. (TXT)

S2 Data Climate data. (TXT)

S1 Fig Temporal sampling intensity in the Forest Inventory Analysis (FIA) and VegBank databases. We show the number of occurrence records within two year bins. We also show the span of elevations occurrence records that were sampled within a given year. For Vegbank, the datasource of the occurrence records is indicated by symbol color.
(TIFF)

S2 Fig Temporal trends in the elevation of occurrence records varies considerably between data sources (i.e., amateur observation, herbarium specimen, professional observation) within a data type (i.e., historical). Each dot represents the elevation and year of observation for occurrence records within the CalFlora database. Each line is the fitted relationship between elevation and year for occurrence records between 1970 and 2009 prior to sample processing (e.g. removing species with insufficient number and temporal timespan of records) when using all records (black line), records reported by amateur botanists (blue line), records of herbarium specimens (green line), records reported by professional botanists (yellow line), and records from field-based methods (red line). For ease in interpreting the data, we do not differentiate data sources for each observation but highlight occurrence data for field-based observations (red dots) and amateur botanist observations (blue dots). Amateur observations increased in elevation over time whereas field-based records increased in elevation until the late 1980’s and then began decreases in the early 2000’s. Data sources are coded accorded to increasing confidence in data (blue: low confidence to red: high confidence).
(TIFF)

S3 Fig Climatic conditions at upper elevational limits tend to be cooler and wetter at higher latitude regions (left side of plot) compared to the lower latitude regions (right side of plot). For each region we show the mean ± 1sd for the mean maximum summer temperature, mean minimum summer temperature, mean summer rain, mean precipitation falling as snow in winter, and mean climate moisture deficit value at the upper distribution limit for the period 1960–2009. Regions are aligned from the most northerly to the most sourtherly.
(TIFF)

S4 Fig Climatic conditions at lower elevational limits tend to be cooler and wetter at higher latitude regions (left side of plot) compared to the lower latitude regions (right side of plot). For each region we show the mean ± 1sd for the mean maximum summer temperature, mean minimum summer temperature, mean summer rain, mean precipitation falling as snow in winter, and mean climate moisture deficit value at the lower distribution limit for the period 1960–2009. Regions are roughly aligned from the most northerly to the most sourtherly.
(TIFF)

S5 Fig Temperatures have warmed in most regions since the 1960s whereas as snowfall has tended to decrease. Each dot represents a climatic measurement for a single species-year combination. Only climate measurements for the upper distribution limits are shown. Climate variables include Tmax: mean maximum summer temperature, Tmin: mean minimum summer temperature, Rain: total summer precipitation, Snow: total winter precipitation falling as snow) and year (1960–2009) for the highest occurrence location of each species within regions. The dashed red line represents the relationship between climate and year across all species within a region.
(TIFF)

S1 Table Correlation amongst the rate of change in climate variables at high and low elevation limits. The rate of change in minimum winter temperatures is not included as it was not significant in any linear models testing which local climatic changes (over the past 50 years explain the direction of elevational distribution shifts at high and low elevation limits over the last 40 years. The rate of change in climate variables is calculated for each species within a region.
(DOCX)

S2 Table The effect of climate variables considered but not included in the final models on raw shift rates. Latitude is not included in the final model as it is correlated with the rate of change in summer minimum temperature (0.709). Longitude and Hargreaves moisture deficit (MoistDef) are not included as they are correlated with the rate of change in summer maximum temperature (0.589, 0.737 respectively). We show the effect of mean Hargreaves moisture deficit (MoistDef), latitude, and longitude on high elevation distribution shift direction when the explanatory variable is considered in the multivariate model and univariate model (alone). We also show the effect of warming maximum and minimum winter temperature (TmaxWt, TminWt) alone. Results are similar for the low elevation distribution limit.
(DOCX)

S3 Table A. AIC values for candidate models explaining the direction that distributions shifted at (A) upper and (B) lower distribution limits. Linear models examining the shift direction (upward or not) of 430 plant species-region combinations between 1970 and 2009 (climate data missing from 14 species-region combinations) at A) upper distribution limits and B) lower distribution limits. The best model, based on the simplest model with the lowest AIC value is in bold for the entire study area (AIC, ΔAIC, 430 species) and the low and high elevation limits. For distribution limits, we show the AIC value for the model with the lowest AIC value and the change in AIC for all other models. K is the number of parameters in the model. Explanatory variables considered include: rate of change in mean maximum and minimum temperatures during the summer (Tmax; Tmin), rate of change in total precipitation falling as rain during the summer (Rain) and snow during the winter (Snow), and mean annual Hargreaves moisture deficit (MoistDef). Winter warming was considered but was not included in the multivariate models as winter warming was a poor predictor of the direction of distribution shifts in univariate models. Maximum winter temperatures warmed for 99% (426/430; climate data missing for 12 species) and minimum winter temperatures warmed for 100% of species (430/430). *na* indicates model not run due to high correlation between parameters. All models included region as a random effect. Explanatory variables were modeled as fixed effects.
(DOCX)

S4 Table Parameter values for best-fit models for the direction that upper and lower distribution limits shifted. Comparison of model results for low (columns 1–4) and high (columns 5–8) elevation limits when distribution shift for 444 plant species-region combinations between 1970 and 2009 is included as a binary variable (upward or not) or as a continuous variable (shift rate). For each model (four models- Direction or Rate at Low or High elevation limits), we provide coefficients and t-values for each explanatory variable. Model formulation is based on the best model based on changes in AIC values (Direction of change shown in S3 Table). Explanatory variables include: rate of change in mean maximum and minimum temperatures during the summer (Tmax; Tmin), rate of change in total precipitation falling as rain during the summer (Rain) and snow during the winter (Snow), and mean annual Hargreaves moisture deficit (MoistDef).
(DOCX)
