# Supplementary material for: Climate Warming and Seasonal Precipitation Change Interact to Limit Species Distribution Shifts across Western North America
Source: PLoS One. 2016 Jul 22;11(7):e0159184. doi: 10.1371/journal.pone.0159184 (PMC4957754; doi:10.1371/journal.pone.0159184)
Supplement: S1 Appendix — (DOCX) [file pone.0159184.s001.docx]

**Supplementary Methods**

*Databases*

In total, we used occurrence records (presences) from five databases with information on vegetation in western North America. We describe the databases here. The databases contain species occurrence records collected primarily using field-based methods (plots, transects, repeat sampling) or historical-based methods (records in herbariums, records from undirected searches). Three databases are primarily field-based (USDA Forest Inventory and Analysis, VegBank, Government of Canada Ecological Site Information database) and two of our databases contain both historical records and field-based records (CalFlora, Global Biodiversity Information Facility).

The USDA Forest Inventory and Analysis (<http://apps.fs.fed.us/fiadb-downloads/datamart.html>) has data available from 1994 onwards – collected using field based efforts (Fig A). Although the number of occurrence records increased significantly after 2004, the minimum and maximum elevations sampled were consistent (4masl ± 12m; 3609masl ± 372m) for the time period 2001-2013 (Fig Ai,ii). Most records are for trees and seedlings, however, occurrence records for herbaceous plants are available for a few states.

The VegBank (<http://vegbank.org>) data included in this study include plot data collected for three projects: the Southwest regional GAP analysis project (<http://earth.gis.usu.edu/swgap>), development of 49 alliances of the US National Vegetation Classification (Composition and function of vegetation alliances in the Interior Northwest, <http://usnvc.org/>), and the National Park Service (<http://www.usgs.gov/core_science_systems/csas/vip/>). Although the number and size of plots varies between projects, all projects provide a list of species present within plots. Further details on methodology on these data sources are available through provided links above or through the VegBank website. Visual inspection of occurrence records indicates that both the number of occurrences and the elevation of occurrence records have increased over time (Fig Aiii,iv). In addition, there are temporal differences between the projects in the maximum elevation of sampling. Earlier records (prior to 1994) come exclusively from the Interior NW dataset. After 1994, data come from the GAP and NPS. GAP and NPS records are collected at higher elevations than the Interior NW (Fig Aiv).

The Government of Canada Ecological Site Information database (<http://srd.alberta.ca/MapsPhotosPublications/Maps/ResourceDataProductCatalogue/Biophysical.aspx>) consists of a compilation of data collected over 20 years from various studies and research programs, including permanent sample plots, stand dynamics studies, and ecological land classification surveys. The number and elevation span of occurrence records and elevation varies between years (Fig Av,vi).

CalFlora ([www.calflora.org](http://www.calflora.org)) is an online database containing plant occurrence observations from 31 different datasets. These datasets differ in their motivations, methodology, and data quality. For instance, occurrences reported by individuals may come with high confidence (iNaturalist research grade, Professional botanist) or lower confidence (observer of any age but not classed as a botanist of any skill level). To account for differences in data quality, we downloaded occurrence records only for datasets where occurrences were observed in the field (not occurrences reported in the literature), were collected by trained staff (i.e. USGS, USDA, BLM, California Department of Fish and Wildlife employees) or professional botanists. Although we were more concerned with accuracy and confidence of occurrence records than in the observation methodology employed, we tried to focus on databases that employed some standardized collection methodology (only the two herbarium datasets employed undirected searches). These datasets include data provided by The Nature Conservancy, USDA Forest Service: Ecology Program, USDA Forest Service: Pacific Northwest Forest Inventory Assessment, USGS Central Mojave Vegetation Mapping Project, Yosemite National Park Resource Inventory, G.F. Hrusa Checklists, California Department of Fish and Wildlife Vegetation Classification and Mapping Program, Bureau of Land Management Eagle Lake Field Office Herbarium, Dean W. Taylor Herbarium Specimens, and East Bay CNPS Rare and Unusual Plants Database. Although a few of the datasets in the CalFlora database are encompassed within the FIA and VegBank databases, we include all datasets with sufficient quality in order to ensure that we are not inadvertently excluding data. We checked for and eliminated records that were duplicated [based on latitude and longitude (rounded to 2 decimal places), year or occurrence, and species name] between databases. Occurrence records are available for all years from 1970 onwards (Fig Avii) at a consistent elevation span (Fig Aviii).

The Global Biodiversity Information Facility ([www.gbif.org](http://www.gbif.org)) is an online database providing global occurrence records collated from over 14,000 datasets. These datasets reflect a wide range of sources: herbarium records, records compiled from the literature, citizen science projects, field data collected by individuals, field-based observations sponsored by government agencies. We removed all records in which the latitude or longitude were not directly recorded, where geospatial issues are known to exist, and where location accuracy is greater than 50 m. We carefully considered notes describing any issues, which are associated with each occurrence record, and removed occurrences with irreconcilable issues related to confidence in geographic location or species. Although the number of occurrence records available in this database has decreased since 2005 (Fig Aix), the elevation span of occurrence records has remained fairly constant over time (Fig Ax).

*Assessing impact of data processing choices on estimated shift rates*

Data from all five databases were merged into a single dataset. Duplicates, based on latitude, longitude, year of occurrence, and species name, were identified and removed. Within each region, we removed any species with less than 50 occurrence records or with insufficient temporal timespan of observations. Shift rates were then calculated for each species-region combination as the slope of the relationship between elevation of occurrence and the year the occurrence was noted. We calculate the shift rate at the mean of the distribution and at the upper and lower elevational distribution limits based on the 5^th^ and 95^th^ quantiles.

We evaluate whether our data processing choices (e.g. minimum number of records, record time span) affected shift rate estimates. We expect more extreme shift rates when sample size is small and lower ability to detect shifts when the record length is short. We used a generalized linear mixed effects model, specifying region as a random effect to assess the significance of any relationships between shift rates at the species within region level and the number of occurrences and the timespan occurrences occurred over. Significant effects of any of these explanatory variables would indicate shift rate estimates are influenced by (for example) sample size and time range of samples. However, no effect was observed based on number of occurrence records (linear mixed effects model, t-value = -0.009, se = 0.003, R^2^ = 0.002; Fig B) or record length (linear mixed effects model, t-value = -0.234, se = 0.073, R^2^ = 4.9 x 10^-7^).

We also considered the impacts of classifying species as shifting or not based on the significance of the slope of the relationship between year and elevation (p < 0.05). Based on this method, the minimum significant shift rate would be 2.89 masl/year, which is greater than the minimum shift rate observed using raw (0.43 masl/year) and corrected shift rates (0.03 masl/year) but similar to the mean value presented by Lenoir *et al.* 2008 (2.94 masl/year). Using slope significance to define whether a distribution shift has occurred likely underestimates the occurrence of distribution shifts.


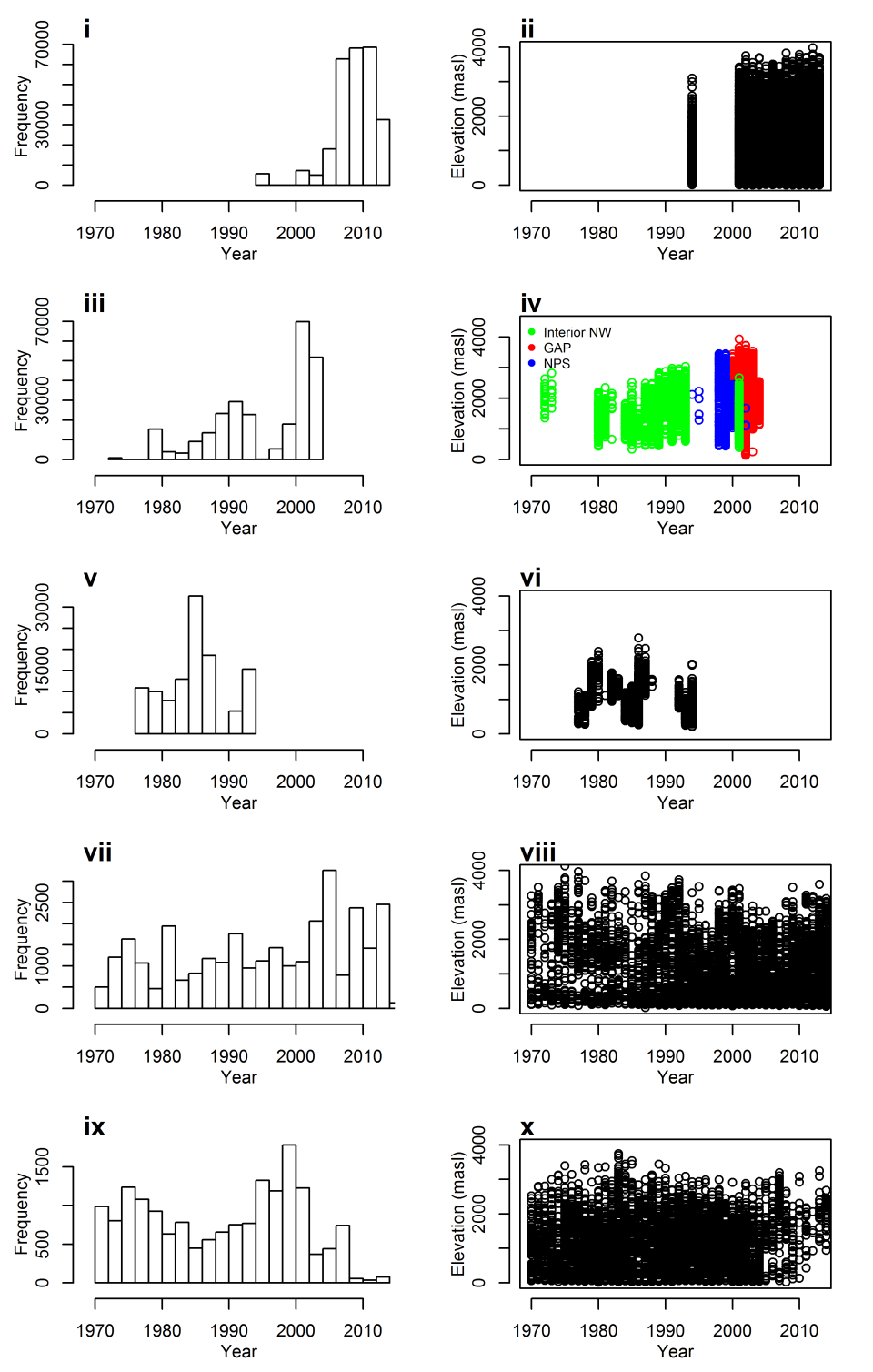


Fig A: Temporal sampling intensity in the Forest Inventory Analysis (FIA) (i,ii), VegBank (iii,iv), Canada (v,vi), CalFlora (vii,viii), and GBIF (ix,x) databases. We show the number of occurrence records within two year bins (i,iii,v,vii,ix). We also show the span of elevations occurrence records were sampled over within a given year (ii,iv,vi,viii,x). For Vegbank (iv), the datasource of the occurrence records is indicated by symbol color.

Fig B: Scatter diagram of annual raw shift rate and i) the number of occurrence records used to calculate raw shift rates and ii) the length of time that observation records span.
