# Supplementary material for: Climate Warming and Seasonal Precipitation Change Interact to Limit Species Distribution Shifts across Western North America
Source: PLoS One. 2016 Jul 22;11(7):e0159184. doi: 10.1371/journal.pone.0159184 (PMC4957754; doi:10.1371/journal.pone.0159184)
Supplement: S2 Appendix — (DOCX) [file pone.0159184.s002.docx]

**Bias Evaluation**

Historical occurrence records, especially herbarium collections but also records obtained from undirected searches (i.e. bioblitz), are likely subject to temporal and spatial biases in collection effort. Although sampling bias is more likely to be present in historical records, it may also be present in occurrence records collected from plots and transects (field-based records). These biases may result from several factors such as non-systematic sampling and differences in sampling-effort between collectors over space and time. It is important to evaluate biases and, if present, either take them into account or correct for them. If biases are present, then estimated species distribution shifts over time may reflect temporal or spatial sampling bias rather than actual distribution shifts. We recognize three primary sources of potential sampling bias in our dataset, bias due to changes in sampling-effort over time, bias due to differences in sampling-effort over latitude or longitude, and bias due to differences in sampling-effort over elevation and time. Sampling biases due to time or latitude and longitude are easily accounted for by excluding or grouping occurrence records. In this study, we do so by grouping and analyzing data within regions.

***Evaluating bias due to changes in sampling effort over time***

Temporal sampling bias is present in our databases, as evident by the increase in the number of occurrence records over time (Fig A). A similar pattern was evident within each region (results not shown). Temporal bias may also result from changes in the accuracy of spatial coordinates and availability of systematically collected occurrence records (plot data). As confidence in coordinate location and the number of occurrence records increase with time (Fig A), we limited our analysis to occurrence records collected from 1970 onwards. Thus, our analyses include those decades when the number of occurrence records was most consistent between years. In fact, the records we analyzed were available annually in all regions except the north and south Canada regions (Fig B). This was primarily due to the frequency in which field samples were collected, roughly 8-year intervals in north Canada, and the absence of historical specimens for the north Canada region.

***Evaluating bias due to differences in sampling-effort over elevation and time***

Evaluating whether bias in elevational sampling effort over time exists is more challenging. Presence-only data provides information on the elevation where species were observed but not the extent of area searched. This means that calculated elevational distribution shifts could reflect changes in species distributions over time (a biological response) or changes in elevation sampling-effort (elevation sampling bias) over time. Although we cannot determine the spatial extent that individual species were searched over, we can evaluate potential influence of elevation sampling bias on species distribution shift estimates. We take several approaches to evaluating these potential biases. First, we evaluated patterns within the data itself for evidence of bias (outlined in the next section). Specifically, we evaluate trends in the relationship between elevation and year for all occurrence records within a region, the baseline trend, and compare the primary direction of distribution shifts (upward, equivocal, downward) by species within regions to this baseline trend. We also compare trends from occurrence records collected using field-based approaches vs. historical specimens to baseline trends. Second, we use null models for individual species by resampling from all samples (outlined in the second section below). Third, we use a jack knifing approach to evaluate the influence of individual years on overall trends (outlined in the third section below). Finally, in the last section, we summarize observations of elevation sampling effort across the study area and between data sources (field vs. historical records). Code for the null model and jack knifing approaches are provided in S3 Appendix.

*Assessment of temporal bias in collection effort using patterns within the data*

We begin our evaluation of patterns within the data itself by calculating the baseline trend of how the elevation of occurrence records has shifted over time. A downward or upward trend would indicate that the elevation over which species were searched has likely decreased or increased over time. Further insights can be gained by comparing the average direction of change (upward, downward, equivocal) of species within regions (Table A) against the baseline trend. If the average direction of change for species is consistent with the slope of the baseline trend (upward, downward, equivocal), it indicates that distribution shift rates may reflect elevation sampling bias or may be amplified by elevation sampling bias. If species distribution shifts are not in the direction of the slope of the baseline trend, it indicates that elevational sampling bias, if present, is dampening our ability to detect distribution shifts, rather than masking them.

Here, we used all unique occurrence records (based on latitude, longitude, elevation, year, and species) post 1970, including those for species with insufficient number or timespan of occurrence records to be included in the final analysis (raw occurrence dataset). By including all species, we are more likely to include the elevation spatial extent that species were searched over. The relationship elevation and year was calculated using linear regression within each region (688 - 14,402 occurrence records per region).

Baseline temporal trends in the elevation of occurrence records (elevation sampling bias) were present in all regions (Fig B, blue lines). Trends were significant (p < 0.05) in all regions but with low R^2^ values (< 0.11). Shifts in sampling effort across all records were consistent with the overall direction that most (>55%) species distributions shifted (raw shift rates) in all regions except the Washington and Utah regions (Fig B, dashed blue lines). In these regions, there was no clear direction that most species were shifting (53% shifting upward) whereas the baseline trends were upward and downward, respectively. Visual inspection of the data suggests that elevation sampling effort has likely changed over time in five of the seven regions.

*Null model evaluation of temporal bias in elevational sampling*

Null models are an effective means to evaluate whether systematic changes in sampling effort have occurred over time, and influenced estimates of shift rates. We follow the methods described in Feely (2012). In this model, we simulate a ‘null set’ of occurrence records (elevation and year) for each species by randomly sampling from our dataset. Specifically, we randomly select from all occurrence records (regardless of species) that were observed within the region and the elevational limits (minimum and maximum ever observed) of the selected species. The number of occurrence records sampled for this ‘null set’ is determined by the observed number of occurrence records for that selected species. We then calculate the ‘null’ shift rate for this simulated ‘null set’ of occurrence records (linear regression between elevation and year). The null shift rate was calculated 500 times (from a different randomly sampled null set of observations). This allowed us to obtain a distribution of null shift rates for each species-region combination (455) within the database. If these null shift rates are significantly different from zero, this implies that there has been a change in elevational sampling-effort over time. This change in elevation sampling effort can be ‘corrected’ for by subtracting the mean null shift rate (representing temporal changes in elevational sampling effort) from the raw shift rate – leaving a ‘corrected’ shift rate.

We found that null shift rates were significant (mean ± sd did not overlap 0) for the Northern Canada, California, Sierra Nevadas, and Montana regions (Fig C). Null shift rates were not significant (mean ± sd overlapped 0) for the Southern Canada, Utah, and Washington regions. Similar results were found for individual species (Fig D). Null shift rates for individual species tended to overlap 0 in Southern Canada, Utah, and Washington regions but not in the other four regions. Thus, based on these null model results, there is indication that elevation sampling-effort has changed significantly over time within the first mentioned regions. Code for the null model analysis is provided in “*S3:* *Statistical Code*”.

*Jack knifing*

We next used a jack knifing approach to evaluate the potential effect of extreme elevation occurrence locations collected in one year (due to accuracy in occurrence record locations or elevation sampling effort) on the relationship between elevation and year in the raw occurrence dataset. To do so, we sequentially remove occurrence records for each year (individually) and re-calculated the relationship between elevation and year (shift rate) for the remaining occurrence records. We then evaluate the slope of the relationship between shift rates with year removed and year for trends. A positive slope would suggest the elevation sampling effort has shifted downhill over time and a negative slope that elevation sampling effort has shifted uphill.

We found that individual years did influence distribution shift rates (Fig E). However, the relationship between shift rates with a year removed and year, although significant (linear model, p < 0.05), had little explanatory power (R^2^ < 0.04) in all regions except California (R^2^ = 0.48; red dashed lines in Fig E). In California, an upward trend in the relationship between shift rates with year removed and year was evident (red line, Fig E). This suggests that elevation sampling effort has shifted downhill over time and is consistent with what was observed in the baseline trends (Fig B) and null model analysis (Fig C). It is worth noting that removing occurrences from a single year changed the value of the shift rate but not the sign, indicating that any year of data alone was not driving directional shifts. Only in one year in one region, south Canada Rockies, did the direction of the slope change when removing records for a particular year. This year was marked by few occurrence records; all at low elevations (see Fig B; SCan year 1993).

A similar effect was observed for individual species (as opposed to all occurrences) (Fig F). For the majority of species (95%), the slope of the relationship between shift rates when year was removed and year was centered on zero (0 ± 0.34 masl). This slope was significant (p < 0.05) for only 0.8% (24/590) of species (Fig F, red dots). No systematic bias in the slope of the relationship between shift rates when year was removed and year according to raw shift rates was evident (Fig F).

*Sampling bias in historical vs. field-based records*

Historical collections are expected to be more prone to sampling bias than field-based collections, but three lines of evidence indicate that historical records were not more prone to sampling bias than field-based records in this data set. First, temporal biases in elevation sampling-effort were as prevalent with field-based records (green lines) as with historical specimens (red lines; Fig B). In all regions except California and south Canada, the slope of relationship between elevation and year was similar between field-based records (Fig B, green lines), historical records (red lines) and the full-combined dataset (blue lines). Second, the percent of species shifting upward and the rate of these shifts within a region were quantitatively similar when using field-based records and historical collections alone, despite differences in temporal trends (Fig G). Finally, even within a data type (historical vs. field-based), the relationship between elevation and year for different data sources can vary considerably. These results highlight that assumptions that one data source is less prone to sampling bias than others should be met with caution.

***Summary of analysis of bias due to differences in sampling-effort over elevation and time***

Temporal bias in elevation sampling effort was evident. The jack knifing approach was the least conservative method (only one region flagged) and the evaluation of spatial patterns was the most conservative approach (five regions flagged). The direction of elevation sampling bias (uphill or downhill over time) was consistent across methods. The southern Canada region was flagged for sampling bias using all three methods and the Utah and Washington regions were flagged in none of the methods.

Analysis of baseline trends in the raw data indicates that bias in elevation sampling-effort over time exists in our dataset. This is clear when viewing the elevation of occurrence records over time (Fig B). It is worth noting that the patterns observed in this dataset, in particular, greater occurrence at higher elevations in the last decade compared to the beginning of the study period, are evident in other databases that are not likely to exhibit temporal biases in elevation sampling-effort (Kelly & Goulden 2008; Lenoir *et al.* 2008). These databases are based on surveys that resample the exact locales of historic sampling locations and use appropriate methods to ensure both presences and absences are characterized. Disentangling whether baseline trends in the raw data reflect bias in elevation sampling-effort or that the majority of species within a region have shifting upward or downward is challenging. Correcting for biases may dampen ability to detect true distribution shifts, yet the consistent result between bias correction methods suggest that sampling bias should be taken into account. We therefore apply a sampling bias correction to our estimated shift rates by subtracting the null model shift rate for each species within a region from the corresponding estimated shift rate.

***An additional method to account for bias, weighting data sources***

Some datasets are known to be more accurate than others. For instance, observations collected by professional botanists rather than amateurs or collected systematically and repeatedly using field-based methods are assumed to hold greater accuracy. Ideally, data from these sources should be given more weight than data sources that were collected by novice or amateur botanists or without a systematic approach. However, these weighting approaches should be viewed with caution. For instance, higher elevation field based sites were added after the 1986 sampling year in the Government of Canada Ecological Site Information database (Fig B in S2 Appendix), thus, weighting field-based records over historical records might introduce a temporal sampling bias. Temporal trends in elevation of observations over time in the CalFlora dataset also suggest weighting field-based records would be problematic (S2 Fig), as there is no indication that data from field-based records are less prone to temporal sampling bias. For instance, both field- (considered most reliable) and herbarium-based (considered least reliable) records show a decline in elevation sampling effort over time whereas a moderately-reliable source (professional botanist observations) and a less-reliable source (amateur botanist observations) both show increases in elevation sampling over time (S2 Fig). In this case, the aggregated dataset appears more reliable than the individual pieces as elevation sampling effort for any given sources (i.e. red or blue dots in S2 Fig) changes over time. The aggregated data show less temporal fluctuations in sampling elevational sampling effort. In summary, we chose to use unweighted data, as weighting data sources is unlikely to be a simple, straight-forward process, and would require considerable thought and methodological development beyond the scope of this study.

|  | NCan | SCan | WA | MT | CA | NV | UT |
| --- | --- | --- | --- | --- | --- | --- | --- |
| **Mean shift rate (masl yr^-1^)** | |  |  |  |  |  |  |
| Raw shift rate | 4.97 | -4.11 | 1.38 | -15.42 | -3.21 | -6.43 | 0.73 |
| Corrected shift rate | 0.37 | 2.07 | -0.12 | -3.16 | 5.73 | 13.13 | 2.51 |
| Upward only (corrected) | 2.33 | 7.02 | 5.64 | 4.93 | 11.33 | 21.28 | 7.31 |
| Downward only (corrected) | -1.94 | -5.96 | -3.96 | -7.13 | -4.68 | -9.51 | -7.78 |
| **Percent shifting upward** |  |  |  |  |  |  |  |
| Distribution mean (raw) | 94.5 | 30.8 | 53.3 | 3.2 | 37.5 | 38.0 | 53.9 |
| Distribution mean (corrected) | 43.6 | 58.9 | 37.8 | 32.0 | 64.1 | 73.5 | 68.2 |
| Upper limit (raw) | 79.6 | 19.8 | 71.1 | 4.7 | 42.5 | 49.3 | 61.5 |
| Upper limit (corrected) | 49.1 | 25.6 | 57.8 | 27.2 | 56.4 | 75.0 | 63.6 |
| Lower limit (raw) | 46.3 | 84.6 | 46.7 | 22.8 | 42.5 | 46.7 | 53.9 |
| Lower limit (corrected) | 3.6 | 93.3 | 38.8 | 54.4 | 64.1 | 82.4 | 59.1 |
| Number of Species | 46 | 86 | 43 | 118 | 28 | 68 | 21 |

**S2 Table A:**  **The mean raw and corrected shift rate and percent of species shifting uphill within regions across western North America.** The mean shift rate is shown across all species using raw and corrected distribution shift rates and for the subset of species that are shifting uphill (higher elevations) or downhill (lower elevations). Shift rates were estimated at the mean of the distribution using both field-based records and historical collections, expect for Northern Canada (NCan), in which only field-based records were available. We show the percent of species shifting upward within a region when slopes are calculated using the mean of the distribution. We also show the results for the upper and lower distribution limits after null model correction.

Fig A: **The number of occurrence records (based on latitude, longitude, elevation) across western USA has increased steadily over the time period 1850 - 2009**. Analysis was limited to occurrence records collected from 1970 onwards (right side of dashed line).


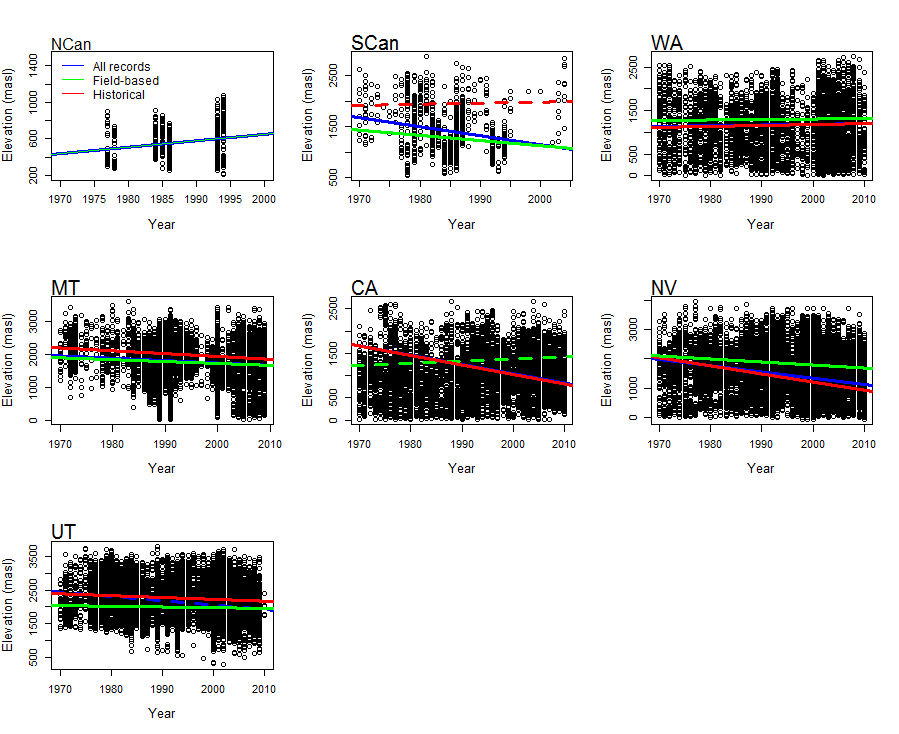
 Fig B: **The elevation over which occurrence records were recorded changed over time in most regions.** Relationship between elevation and year for all occurrence records between 1970 and 2009 prior to sample processing (e.g. removing species with insufficient number and temporal timespan of records) within a region when using all records (blue line), field-based records alone (green line), and historical collections alone (red line). Dashed lines indicate that the direction of the relationship is inconsistent with the direction (upward, equivocal, downward) that most (>55%) species within the region are shifting when calculated using all records, field-based records, or historical specimens after removing species with less than 50 records.

Fig C: **Temporal bias in elevation sampling effort (demonstrated by 95% confidence intervals not overlapping 0) are just as likely at high and low temporal trends in elevation occurrences.** Temporal trends in elevation occurrences is calculated as the slope of the relationship between elevation and year of all occurrence records within a region. Each dot and line represents the mean and 95% confidence interval for species null distribution shift rates within a region according to the temporal trends in elevation occurrences for that region. Null species distribution shift rates are limited to those species with sufficient number (>50) and timespan of occurrence records.

Fig D: **Null distribution shifts were significant for all or most species in MT, NCan, CA, and NV, suggesting that the presence temporal bias in elevation sampling effort in these regions.** For each species within each region we show the range of null distribution shifts (mean ±1 sd). Null distribution shift rates are calculated from 500 estimates of distribution shift rate simulations using pooled datasets across all species. The null distribution was insignificant for all species in UT and for most species in SCan and WA.

Fig E: **Jack knifing approach indicates elevation sampling bias is likely present in the California region but not in the other regions.** Each dot indicates the estimate shift when then occurrence records from a given year (x-axis) are removed. Analysis is done within a region and includes all species, including those with less than 50 occurrence records. Dashed lines represent the estimated rate shift rate (no occurrences are removed). Red lines represent the relationship between all estimated shift rates when occurrences are removed and year.

Fig F: **Temporal bias in elevation sampling effort, based on jack knifing approach, was not evident for individual species within regions.** Each open circle is temporal effect (slope) of removing occurrence data from a single year on estimated raw shift rates (equivalent to red line in Fig. S11) (y-axis) and the raw shift rate (x-axis). Only species with at least 50 occurrence records are shown. Red symbols reflect estimated slopes that are significant to p < 0.05.

Fig G: **The mean direction and rate of that distribution means have shifted are similar between records obtained from historical and field-based methods.** For the unsubsetted data we present i) the percent of species shifting upward within a region and ii) rate of shift (with standard error bars). Dark bars reflect result for data from historical collections and light bars from field-based records. Dashed lines reflect 50% of species within a region shifting upward at rates greater than 0.5 m/yr. Insufficient number of historical records were available for analysis for northern Canada (NCan).

**References:**

Feeley K.J. (2012). Distributional migrations, expansions, and contractions of tropical plant species as revealed in dated herbarium records. *Global Change Biology*, 18, 1335-1341.

Kelly A.E. & Goulden M.L. (2008). Rapid shifts in plant distribution with recent climate change. *PNAS*, 105, 11823–11826.

Lenoir J., Gégout J.C., Marquet P.A., de Ruffray P. & Brisse H. (2008). A significant upward shift in plant species optimum elevation during the 20th century. *Science*, 320, 1768-1771.
