# Supplementary figures and images for: Climate Warming and Seasonal Precipitation Change Interact to Limit Species Distribution Shifts across Western North America
Source: PLoS One. 2016 Jul 22;11(7):e0159184. doi: 10.1371/journal.pone.0159184 (PMC4957754; doi:10.1371/journal.pone.0159184)

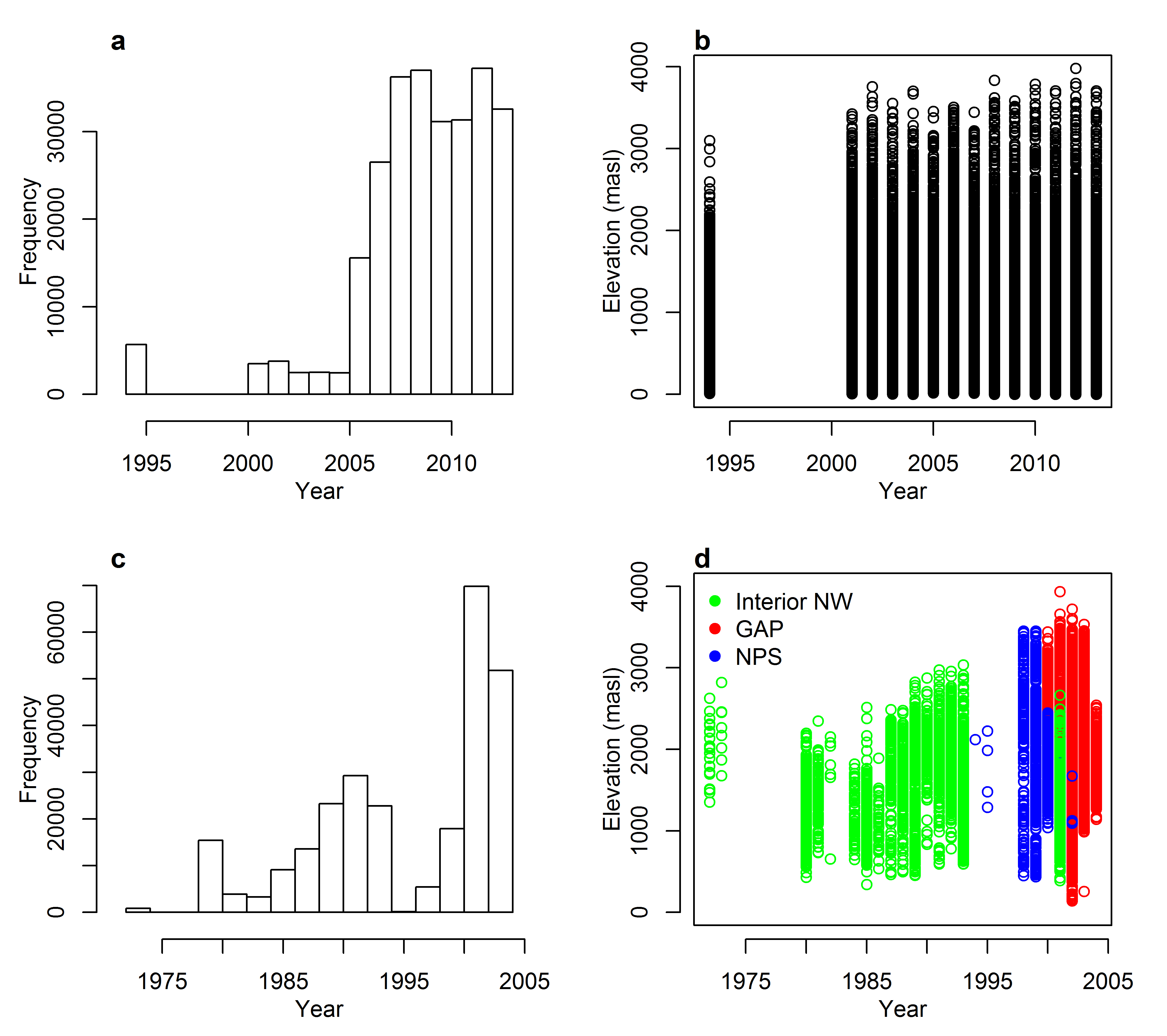

Supplement: S1 Fig — We show the number of occurrence records within two year bins. We also show the span of elevations occurrence records that were sampled within a given year. For Vegbank, the datasource of the occurrence records is indicated by symbol color. (TIFF) [file pone.0159184.s006.tiff]

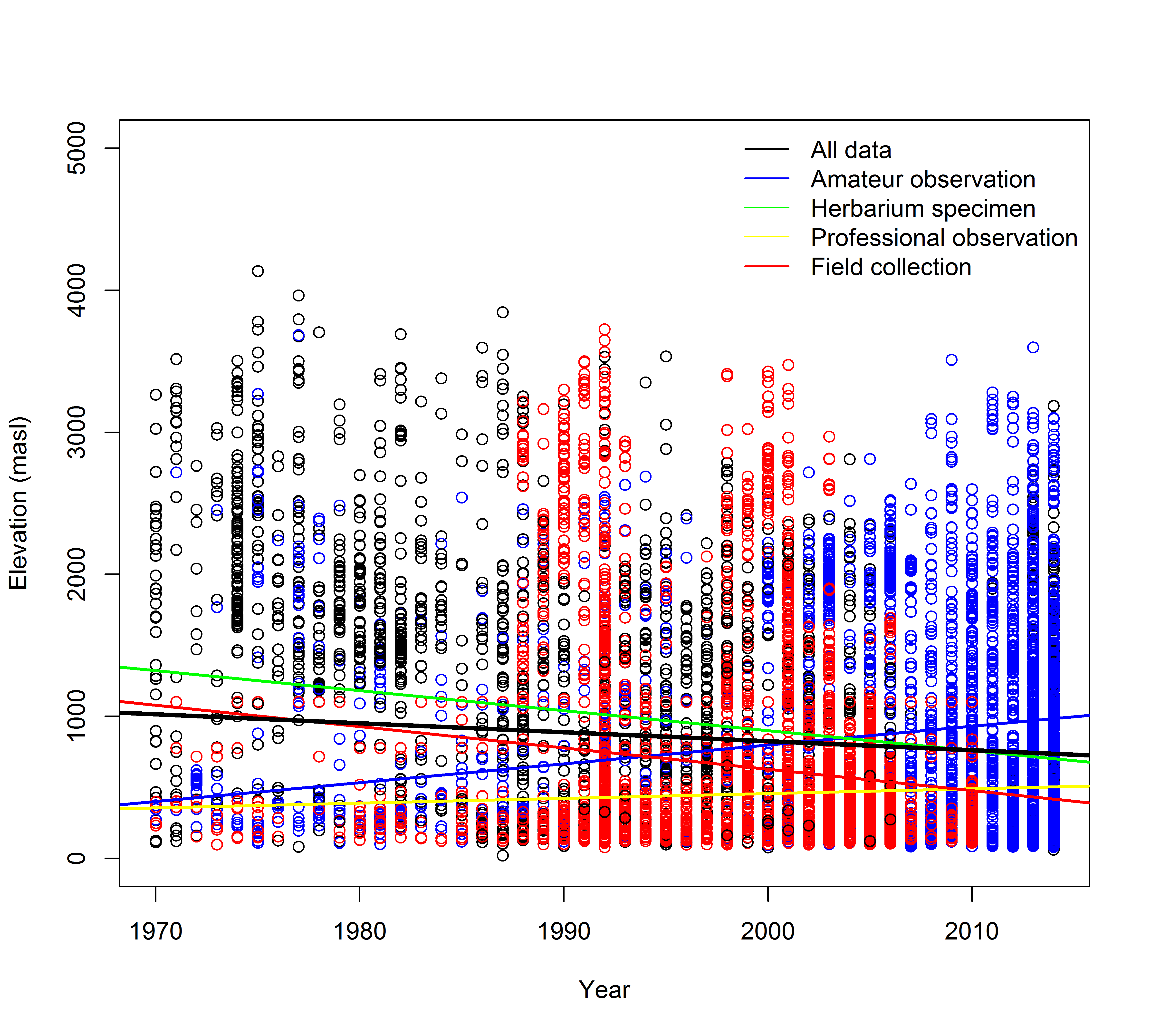

Supplement: S2 Fig — Each dot represents the elevation and year of observation for occurrence records within the CalFlora database. Each line is the fitted relationship between elevation and year for occurrence records between 1970 and 2009 prior to sample processing (e.g. removing species with insufficient number and temporal timespan of records) when using all records (black line), records reported by amateur botanists (blue line), records of herbarium specimens (green line), records reported by professional botanists (yellow line), and records from field-based methods (red line). For ease in interpreting the data, we do not differentiate data sources for each observation but highlight occurrence data for field-based observations (red dots) and amateur botanist observations (blue dots). Amateur observations increased in elevation over time whereas field-based records increased in elevation until the late 1980’s and then began decreases in the early 2000’s. Data sources are coded accorded to increasing confidence in data (blue: low confidence to red: high confidence). (TIFF) [file pone.0159184.s007.tiff]

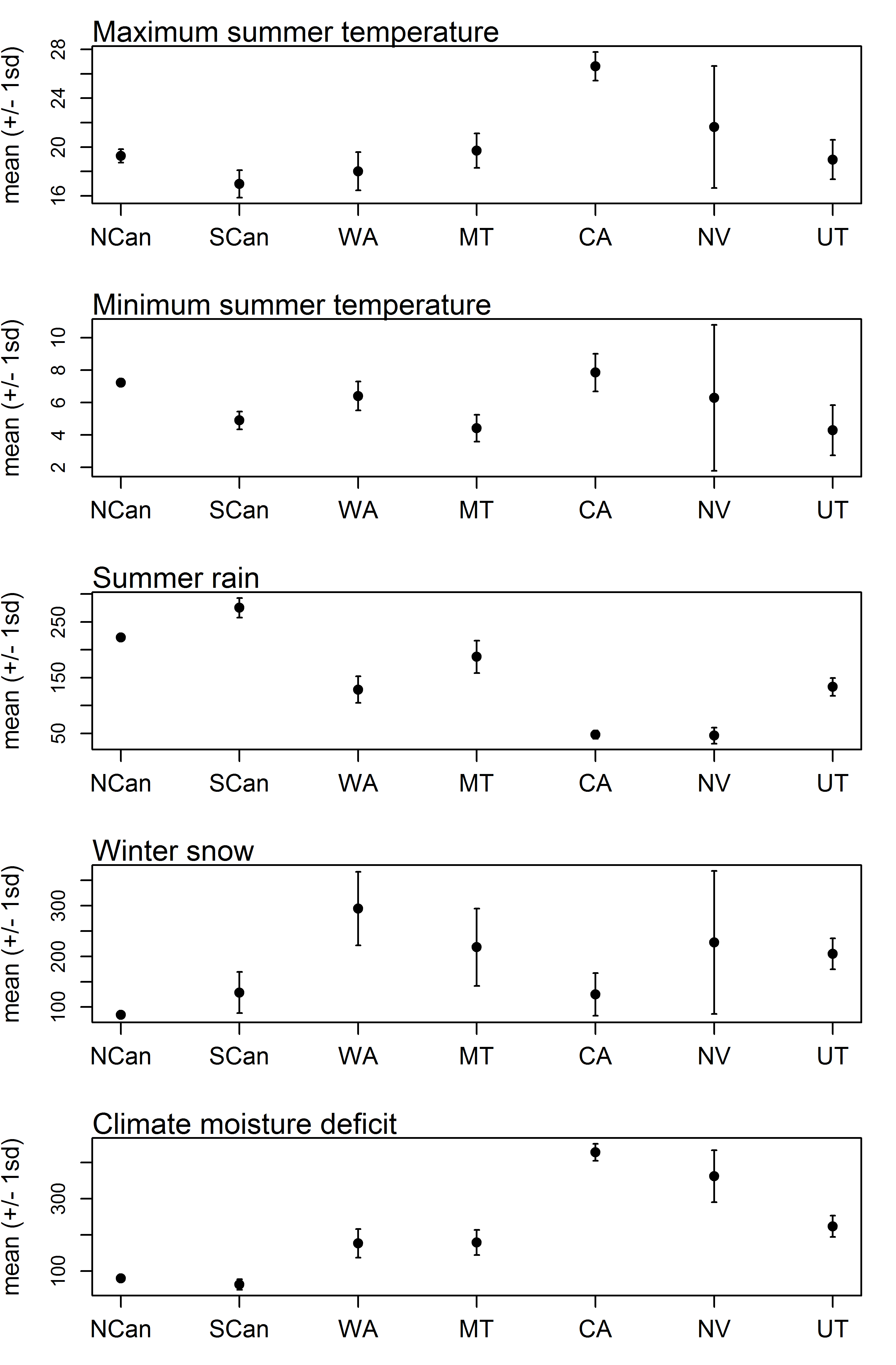

Supplement: S3 Fig — For each region we show the mean ± 1sd for the mean maximum summer temperature, mean minimum summer temperature, mean summer rain, mean precipitation falling as snow in winter, and mean climate moisture deficit value at the upper distribution limit for the period 1960–2009. Regions are aligned from the most northerly to the most sourtherly. (TIFF) [file pone.0159184.s008.tiff]

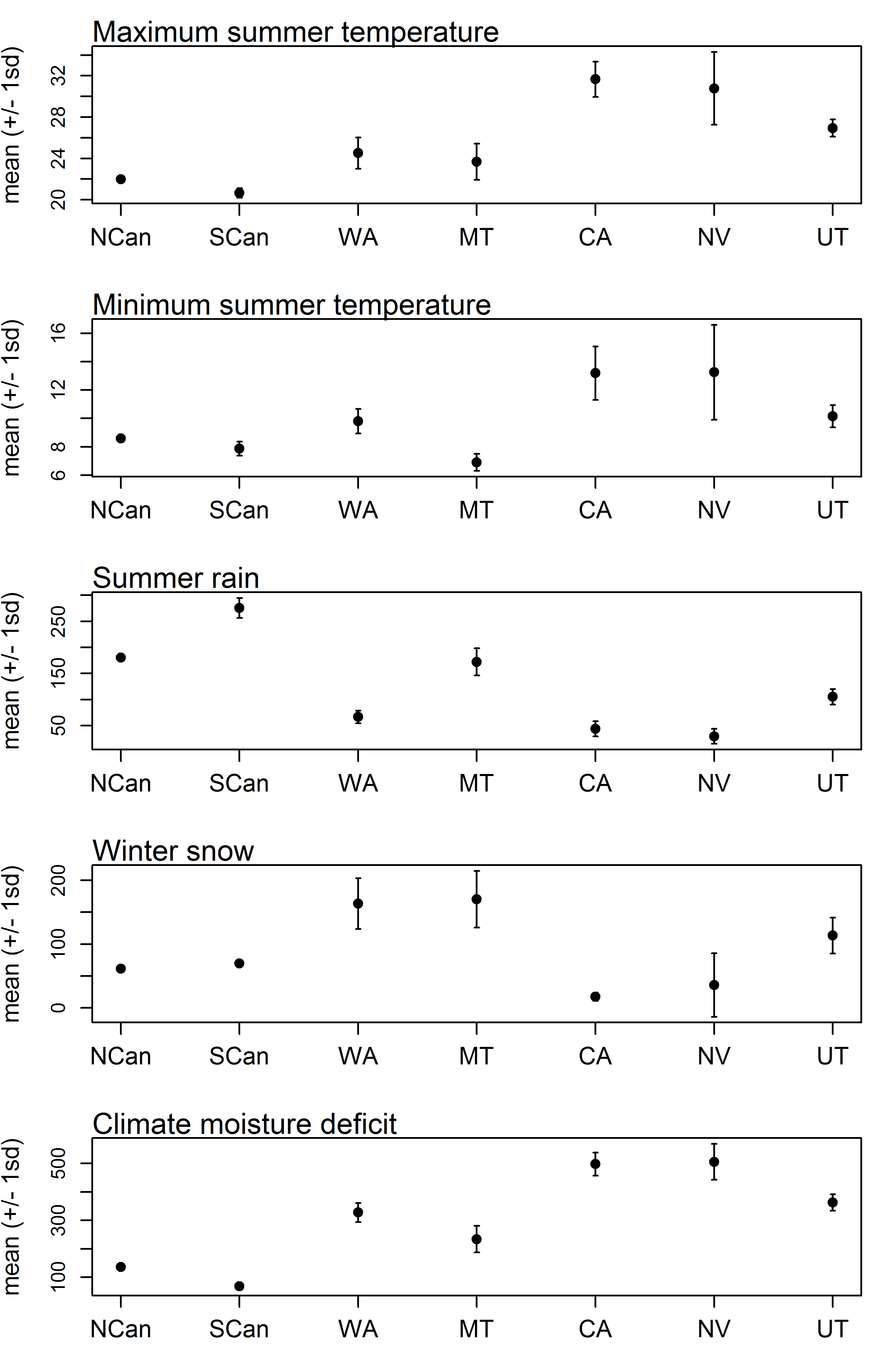

Supplement: S4 Fig — For each region we show the mean ± 1sd for the mean maximum summer temperature, mean minimum summer temperature, mean summer rain, mean precipitation falling as snow in winter, and mean climate moisture deficit value at the lower distribution limit for the period 1960–2009. Regions are roughly aligned from the most northerly to the most sourtherly. (TIFF) [file pone.0159184.s009.tiff]

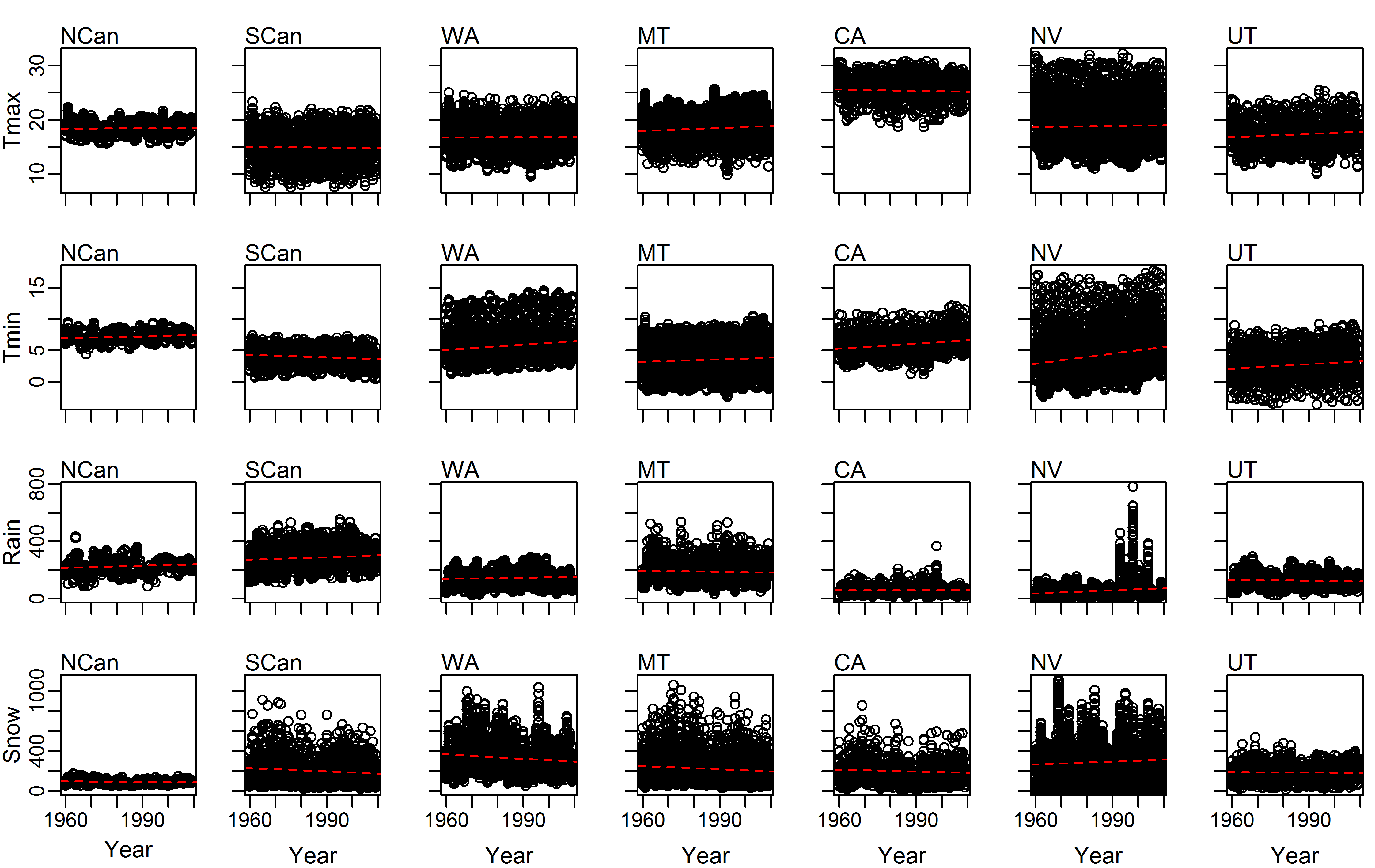

Supplement: S5 Fig — Each dot represents a climatic measurement for a single species-year combination. Only climate measurements for the upper distribution limits are shown. Climate variables include Tmax: mean maximum summer temperature, Tmin: mean minimum summer temperature, Rain: total summer precipitation, Snow: total winter precipitation falling as snow) and year (1960–2009) for the highest occurrence location of each species within regions. The dashed red line represents the relationship between climate and year across all species within a region. (TIFF) [file pone.0159184.s010.tiff]
