# Supplementary material for: Climate Warming and Seasonal Precipitation Change Interact to Limit Species Distribution Shifts across Western North America
Source: PLoS One. 2016 Jul 22;11(7):e0159184. doi: 10.1371/journal.pone.0159184 (PMC4957754; doi:10.1371/journal.pone.0159184)
Supplement: S1 Table — The rate of change in minimum winter temperatures is not included as it was not significant in any linear models testing which local climatic changes (over the past 50 years explain the direction of elevational distribution shifts at high and low elevation limits over the last 40 years. The rate of change in climate variables is calculated for each species within a region. (DOCX) [file pone.0159184.s011.docx]

Upper Limits

|  |  | Summer |  |  | Winter |  |
| --- | --- | --- | --- | --- | --- | --- |
|  |  | Temperature  Maximum | Temperature  Minimum | Rainfall | Temperature  Maximum | Snowfall |
| Summer | Temperature Maximum |  | 0.04 | -0.24 | 0.51 | -0.14 |
|  | Temperature Minimum | 0.04 |  | 0.81 | -0.45 | 0.53 |
|  | Rainfall | -0.24 | 0.81 |  | -0.34 | 0.43 |
| Winter | Temperature Maximum | 0.51 | -0.45 | -0.34 |  | -0.32 |
|  | Snowfall | -0.14 | 0.53 | 0.43 | -0.32 |  |

Lower Limits

|  |  | Summer |  |  | Winter |  |
| --- | --- | --- | --- | --- | --- | --- |
|  |  | Temperature  Maximum | Temperature  Minimum | Rainfall | Temperature  Maximum | Snowfall |
| Summer | Temperature Maximum |  | 0.49 | -0.04 | -0.16 | 0.06 |
|  | Temperature Minimum | 0.49 |  | 0.47 | -0.51 | 0.71 |
|  | Rainfall | -0.04 | 0.47 |  | 0.20 | 0.12 |
| Winter | Temperature Maximum | -0.16 | -0.51 | 0.20 |  | -0.57 |
|  | Snowfall | 0.06 | 0.74 | 0.12 | -0.57 |  |

**S2 Table:** **Correlation amongst the rate of change in climate variables at upper and lower distribution limits.** The rate of change in minimum winter temperatures is not included as it was not significant in any linear models testing which local climatic changes (over the past 50 years explain the direction of elevational distribution shifts at high and low elevation limits over the last 40 years. The rate of change in climate variables is calculate for each species within a region.
