# Supplementary material for: Climate Warming and Seasonal Precipitation Change Interact to Limit Species Distribution Shifts across Western North America
Source: PLoS One. 2016 Jul 22;11(7):e0159184. doi: 10.1371/journal.pone.0159184 (PMC4957754; doi:10.1371/journal.pone.0159184)
Supplement: S2 Table — Latitude is not included in the final model as it is correlated with the rate of change in summer minimum temperature (0.709). Longitude and Hargreaves moisture deficit (MoistDef) are not included as they are correlated with the rate of change in summer maximum temperature (0.589, 0.737 respectively). We show the effect of mean Hargreaves moisture deficit (MoistDef), latitude, and longitude on high elevation distribution shift direction when the explanatory variable is considered in the multivariate model and univariate model (alone). We also show the effect of warming maximum and minimum winter temperature (TmaxWt, TminWt) alone. Results are similar for the low elevation distribution limit. (DOCX) [file pone.0159184.s012.docx]

**S1 Table**

|  |  | Multivariate model | | | Univariate model | | |
| --- | --- | --- | --- | --- | --- | --- | --- |
|  |  | Estimate | SE | t-value | Estimate | SE | t-value |
|  | **MoistDef** | -0.182 | 0.112 | -1.629 | -0.167 | 0.101 | -1.658 |
|  | **Latitude** | -0.015 | 0.010 | -1.535 | -0.008 | 0.010 | -0.777 |
|  | **Longitude** | -0.012 | 0.014 | -0.857 | -0.014 | 0.014 | -1.007 |
|  | **TmaxWt** |  |  |  | -3.430 | 3.258 | -1.053 |
|  | **TminWt** |  |  |  | -0.043 | 0.052 | -0.820 |

The effect of climate variables considered but not included in the final models. Latitude is not included as it is correlated with the rate of change in summer minimum temperature (0.709). Longitude and Hargreaves moisture deficit (MoistDef) are not included as they are correlated with the rate of change in summer maximum temperature (0.589, 0.737 respectively). We show the effect of mean Hargreaves moisture deficit (MoistDef), latitude, and longitude on high elevation distribution shift direction when the explanatory variable is considered in the multivariate model and univariate model (alone). We also show the effect of warming maximum and minimum winter temperature (TmaxWt, TminWt) alone. Results are similar for the low elevation distribution limit.
