# Supplementary material for: Climate Warming and Seasonal Precipitation Change Interact to Limit Species Distribution Shifts across Western North America
Source: PLoS One. 2016 Jul 22;11(7):e0159184. doi: 10.1371/journal.pone.0159184 (PMC4957754; doi:10.1371/journal.pone.0159184)
Supplement: S3 Table — Linear models examining the shift direction (upward or not) of 430 plant species-region combinations between 1970 and 2009 (climate data missing from 14 species-region combinations) at A) upper distribution limits and B) lower distribution limits. The best model, based on the simplest model with the lowest AIC value is in bold for the entire study area (AIC, ΔAIC, 430 species) and the low and high elevation limits. For distribution limits, we show the AIC value for the model with the lowest AIC value and the change in AIC for all other models. K is the number of parameters in the model. Explanatory variables considered include: rate of change in mean maximum and minimum temperatures during the summer (Tmax; Tmin), rate of change in total precipitation falling as rain during the summer (Rain) and snow during the winter (Snow), and mean annual Hargreaves moisture deficit (MoistDef). Winter warming was considered but was not included in the multivariate models as winter warming was a poor predictor of the direction of distribution shifts in univariate models. Maximum winter temperatures warmed for 99% (426/430; climate data missing for 12 species) and minimum winter temperatures warmed for 100% of species (430/430). na indicates model not run due to high correlation between parameters. All models included region as a random effect. Explanatory variables were modeled as fixed effects. (DOCX) [file pone.0159184.s013.docx]

**S2 Table. AIC values for candidate models explaining directional distribution shifts**

**Table A**

| Model Name | K | Upper limit |
| --- | --- | --- |
| Tmin*Snow + Tmax + Rain | 7 | 537 |
| Tmax*Snow + Tmin + Rain | 7 | 542 |
| Tmax +Tmin + Snow + Rain | 6 | 542 |
| Tmax*Snow + Tmin | 6 | 541 |
| Tmax +Tmin + Snow | 5 | 542 |
| Tmax*Snow | 5 | 551 |
| Tmin*Snow | 5 | **536** |
| Tmin + Snow | 4 | 550 |

**Table B**

| Model Name | K | Lower limit |
| --- | --- | --- |
| Tmax*Snow + Rain + MoistDef | 7 | 429 |
| Tmax*Rain + Snow + MoistDef | 7 | 429 |
| Tmin* Rain + Snow + Tmax | 7 | 431 |
| Tmax* Rain + Snow + Tmin | 7 | 430 |
| Tmax +Tmin + Snow + Rain | 6 | 432 |
| Tmax*Rain + MoistDef | 6 | 428 |
| Tmax*Snow + Tmin | 6 | 428 |
| Tmax +Tmin + Snow | 5 | 430 |
| Tmax*Rain | 5 | **426** |
| Tmin*Rain | 5 | 427 |
| Tmin + Rain | 4 | 428 |

Linear models examining the pattern of distribution shift of 430 plant species-region combinations between 1970 and 2009 (climate data missing from 12 species-region combinations) at A) upper distribution limits and B) lower distribution limits. The best model, based on the simplest model with the lowest AIC value is in bold for the entire study area (AIC, ΔAIC, 430 species) and the low and high elevation limits. For distribution limits, we show the AIC value for the model with the lowest AIC value and the change in AIC for all other models. Explanatory variables considered include: rate of change in mean maximum and minimum temperatures during the summer (Tmax; Tmin), rate of change in total precipitation falling as rain during the summer (Rain) and snow during the winter (Snow), and mean annual Hargreaves moisture deficit (MoistDef). Winter warming was considered but was not included in the multivariate models as winter warming was a poor predictor of the direction of distribution shifts in univariate models. Maximum winter temperatures warmed for 99% (426/430; climate data missing for 12 species) and minimum winter temperatures warmed for 100% of species (430/430). All models included region as a random effect. Explanatory variables were modeled as fixed effects.
