# Supplementary material for: Climate Warming and Seasonal Precipitation Change Interact to Limit Species Distribution Shifts across Western North America
Source: PLoS One. 2016 Jul 22;11(7):e0159184. doi: 10.1371/journal.pone.0159184 (PMC4957754; doi:10.1371/journal.pone.0159184)
Supplement: S4 Table — Comparison of model results for low (columns 1–4) and high (columns 5–8) elevation limits when distribution shift for 444 plant species-region combinations between 1970 and 2009 is included as a binary variable (upward or not) or as a continuous variable (shift rate). For each model (four models- Direction or Rate at Low or High elevation limits), we provide coefficients and t-values for each explanatory variable. Model formulation is based on the best model based on changes in AIC values (Direction of change shown in S3 Table). Explanatory variables include: rate of change in mean maximum and minimum temperatures during the summer (Tmax; Tmin), rate of change in total precipitation falling as rain during the summer (Rain) and snow during the winter (Snow), and mean annual Hargreaves moisture deficit (MoistDef). (DOCX) [file pone.0159184.s014.docx]

**S3 Table. Parameter values for best-fit models for the direction that upper and lower distribution limits shifted.**

| Low elevation limit | |  | | High elevation limit | |  | |
| --- | --- | --- | --- | --- | --- | --- | --- |
|  |  | Coefficient | t-value |  |  | Coefficient | t-value |
| Tmax*Rain | Direction | 0.061 | 2.280 | Tmin*Snow | Direction | 0.098 | 3.244 |
|  | Rate | 3.542 | 3.953 |  | Rate | 5.072 | 4.031 |
| Snow | Direction | 0.141 | 2.060 |  |  |  |  |
|  | Rate | 6.327 | 2.811 |  |  |  |  |
| MoistDef | Direction | -0.143 | -1.290 |  |  |  |  |
|  | Rate | -6.586 | -1.835 |  |  |  |  |

Comparison of model results for low (columns 1-4) and high (columns 5-8) elevation limits when distribution shift for 444 plant species-region combinations between 1970 and 2009 is included as a binary variable (direction of change) or a continuous variable (shift rate). For each model (four models), we provide coefficients and t-values for each explanatory variable. Model formulation is based on the best model based on changes in AIC values (Table S2). Explanatory variables include: rate of change in mean maximum and minimum temperatures during the summer (Tmax; Tmin), rate of change in total precipitation falling as rain during the summer (Rain) and snow during the winter (Snow), and mean annual Hargreaves moisture deficit (MoistDef).
